# Non-communicable diseases in sub-Saharan Africa: a scoping review of large cohort studies

**DOI:** 10.7189/jogh.09.020409

**Published:** 2019-12

**Authors:** Kathleen Mudie, Melisa Mei Jin, Tan, Lindsay Kendall, Juliet Addo, Isabel dos-Santos-Silva, Jennifer Quint, Liam Smeeth, Sarah Cook, Dorothea Nitsch, Barnabas Natamba, Francesc Xavier Gomez-Olive, Agbor Ako, Pablo Perel

**Affiliations:** 1 Department of Non-Communicable Disease Epidemiology, London School of Hygiene and Tropical Medicine, London, UK; 2 Saw Swee Hock School of Public Health, National University of Singapore, Singapore; 3 Biostatistics, GlaxoSmithKline, Stevenage, UK; 4 Africa NCD Open Lab, Global Health Catalyst, GlaxoSmithKline, Stevenage, UK; 5 Population and Occupational Disease, National Heart and Lung Institute, Imperial College London, London, UK; 6 MRC/UVRI and LSHTM Uganda Research Unit; 7 MRC/Wits Rural Public Health and Health Transitions Research Unit (Agincourt), School of Public Health, University of the Witwatersrand, South Africa

## Abstract

**Background:**

Non-communicable diseases (NCDs) cause a large and growing burden of morbidity and mortality in sub-Saharan Africa. Prospective cohort studies are key to study multiple risk factors and chronic diseases and are crucial to our understanding of the burden, aetiology and prognosis of NCDs in SSA. We aimed to identify the level of research output on NCDs and their risk factors collected by cohorts in SSA.

**Methods:**

We conducted a scoping review to map the extent of current NCDs research in SSA by identifying studies published after the year 2000 using prospectively collected cohort data on any of the six NCDs (cardiovascular diseases, diabetes, obesity, chronic kidney disease, chronic respiratory diseases, and cancers), ≥1 major risk factor (other than age and sex), set only within SSA, enrolled ≥500 participants, and ≥12 months of follow-up with ≥2 data collection points (or with plans to). We performed a systematic search of databases, a manual search of references lists from included articles and the INDEPTH network website, and study investigators from SSA were contacted for further articles.

**Results:**

We identified 30 cohort studies from the 101 included articles. Eighteen countries distributed in West, Central, East and Southern Africa, were represented. The majority (27%) set in South Africa. There were three studies including children, twenty with adults, and seven with both. 53% of cohorts were sampled in general populations, 47% in clinical populations, and 1 occupational cohort study. Hypertension (n = 23) was most commonly reported, followed by obesity (n = 16), diabetes (n = 15), CKD (n = 6), COPD (n = 2), cervical cancer (n = 3), and breast cancer (n = 1). The majority (n = 22) reported data on at least one demographic/environmental, lifestyle, or physiological risk factor but these data varied greatly.

**Conclusions:**

Most studies collected data on a combination of hypertension, diabetes, and obesity and few studies collected data on respiratory diseases and cancer. Although most collected data on different risk factors the methodologies varied greatly. Several methodological limitations were found including low recruitment rate, low retention rate, and lack of validated and standardized data collection. Our results could guide potential collaborations and maximize impact to improve our global understanding of NCDs (and their risk factors) in SSA and also to inform future research, as well as policies.

The pattern of disease occurrence in sub-Saharan Africa (SSA) is changing and the significance of chronic diseases is increasing [1]. Non-communicable diseases (NCDs) cause a large and growing burden of death and disability in SSA; with regional hypertension prevalence of 48%, diabetes of 5.1%, and obesity of 20% [2]. It is projected that they will overtake infectious diseases as major sources of morbidity and mortality by 2030[3]. Presently, there is still opportunity to introduce measures that would reduce the burden of NCDs in SSA. Currently, SSA lacks adequate data and resources regarding NCDs to respond to this problem [3].

There is no reason to doubt that changes in lifestyle and unplanned, or unsustainable, urbanisation will have adverse health effects in SSA as in other regions. However, due to the combined effects of under-nutrition, infectious diseases, and other environmental factors, NCDs findings from other settings may not be applicable to this region. For example, infectious diseases such as HIV and tuberculosis have been shown to be risk factors for cardiovascular diseases and diabetes, in addition to early life malnutrition. The high prevalence of these potential risk factors in SSA might allow to shed light and understand better some of these associations [4]. There is a need for reliable data about the distribution of NCDs and known risk factors, how they change over time, and how risk factors relate to NCDs outcomes. With reliable data, it is possible to devise effective, long-term prevention strategies to combat disease burden.

Prospective cohort studies have traditionally been used to study multiple risk factors and complex diseases and are crucial to our understanding of the aetiology and prognosis of NCDs within populations [5]. They can be used to study population-specific disease burden, genetic heterogeneity, and geographic and social diversity, which could prove to be novel risk factors. Large and well-designed cohort studies are essential for informing epidemiology, health services, and policies, as well as to stimulate health-promoting change. Due to the limited funding for establishing NCD cohorts in SSA, it is important to map out existing cohorts for primary prevention and prognosis, of which the basis is surveillance and involves the systematic collection of high quality data [6].

Scoping reviews are used to map the extent of existing data available and to identify any gaps in the research [7]. A scoping review from existing large cohorts of NCDs in SSA can answer what is known and what is not known in the region. This scoping review could extend the capacity of existing studies by synthesizing ongoing research initiatives, facilitating consistency in data collection, and providing guidance on what further research would be a beneficial addition to the global understanding of NCDs in the region.

Previous reviews evaluated existing cohorts in SSA but, unlike our review, they were more limited in scope, focusing on either cardiovascular diseases, collection and storage of biological sample data, or on HIV/AIDS patients after antiretroviral therapy (ART) [8-10]. In this scoping review, our main objective was to identify the level of research output on NCDs (cardiovascular disease, diabetes, obesity, respiratory disease, cancer, and chronic kidney disease), as well as their risk and prognostic factors, from large NCDs cohort studies in SSA. The final aim is to identify any limitations and gaps and inform future research.

## METHODS

### Eligibility criteria

**Inclusion criteria**
- Study designs: Prospective cohort studies.
- Study settings: Studies conducted within any SSA country.
- Study publication year: It has been shown that the almost flat number of publications per year in West African countries starts to slowly rise after 2000 and so we chose studies published from 2000 onwards [11]. This does not, however, pertain to when the studies were conducted.
- Study participants: There was no age limitation so that we were not restricted from looking across generations for clues to current patterns of health and disease; a life-course approach was taken.
- Study size: Studies with at least 500 participants.
- NCD data: We included studies which have collected data on at least one of the following NCDs: cardiovascular disease (CVDs), type 2 diabetes, obesity, chronic respiratory diseases, cancer, and chronic kidney disease (CKD). Because some of these NCDs encompass a large number of diseases, we narrowed our scope using expert opinion of SSA study investigators to the following: CVDs to hypertension; respiratory disease to asthma and chronic obstructive pulmonary disease (COPD); and cancers to breast, cervical, prostate, and oesophageal cancers.
- Study data collection points: We chose studies that collected NCD data at baseline and at least once more at follow-up.
- Study follow-up: We included studies with a minimum of 12 months of follow-up. We also included studies that have just started and therefore do not yet have the follow up but have 12 months or more follow-up planned.
- There was no language restriction.

**Exclusion criteria**
- Studies conducted among populations of SSA origin but residing outside of SSA (eg, migrant studies).
- Studies including a mixed population from inside and outside SSA.
- Studies without at least one major risk factor other than sex and age measured at baseline.

### Types of sources

#### Electronic sources

We systematically searched PubMed, MEDLINE, AfricaPortal, and SCOPUS databases, as well as performed an informal search of Google Scholar, for studies published from 1 January 2000 to 1 March 2018. The electronic database search was conducted using a predefined comprehensive and sensitive search strategy in order to obtain the maximum possible number of studies.

#### Reference lists

We manually searched the reference lists of all relevant citations, as well as references quoted on the INDEPTH network website [12], to identify further articles of interest.

#### Grey literature

We explored UK and US funded cohorts and cohort research databases (ie, UK Cohort Directory, LMIC LPS Directory, Birthcohorts.net, and Global Burden of disease).

### Search strategy

For our search strategy, we used a modified four-step search strategy recommended for all Joanna Briggs Institute (JBI) review types [7,13]. The first step was an initial limited search of two of the online databases mentioned previously. From this search, we ascertained text words contained in the titles and abstracts of identified papers, as well as index terms used to describe the articles. We also sought input from a librarian to assist further with identifying MeSH headings and keywords pertinent to this study. Having identified keywords and index terms, a second search was performed across all included databases using these words. Table S1 in **Online Supplementary Document** presents the search terms used for MEDLINE; adapted in minor ways for other databases. The records were directly exported into a reference management software to facilitate collaboration between investigators during the process. Duplicate records were excluded.

The search strategy was unable to distinguish between cross-sectional studies and cross-sectional analyses of cohort data. We were interested in the cohorts, rather than the published analyses. Two investigators (KM, MMJT) independently screened abstracts of records identified through database searching to exclude articles analysing cross-sectional data only. Any inconsistencies or disagreements were resolved by consensus, or by consultation of a third author (PP), when resolution could not be achieved.

The third step was to obtain the full-text and assess articles for eligibility. When the full-text was unavailable, corresponding authors were contacted. Two investigators (KM, MMJT) independently selected articles that met the inclusion criteria. Any articles that were excluded were listed separately, with reasons for their exclusion. Disagreements were resolved by consultation of a third author (PP). One investigator then manually searched the reference lists of articles identified through database searching.

The fourth and final step was unique to this review - we contacted study investigators from Health & Demographic Surveillance Systems (HDSSs) in SSA to help identify further cohort studies in West Africa (Benin, Burkina Faso, Ivory Coast, Guinea, Mali, Niger, Nigeria, Senegal, and Togo).

### Data extraction

A data extraction form was developed and pre-piloted to ensure all relevant data were extracted (Table S2 in **Online Supplementary Document**). From the included articles, we identified individual cohort studies. For cohort studies with more than one identified article, the most recent and comprehensive publication was used [7]. Study characteristics extracted included country, design, start date, number of data collection points, and main focus (reason for its inception). The funding source of the study and whether the authors reported the potential to access the data was also collected. Data extracted relating to the design of the study included the following: sampling frame and method of sampling, inclusion/exclusion criteria, number eligible, number enrolled, and sample size at final or most recent evaluation. The age, sex, ethnicity, and rural/urban location of participants were extracted, as well as any reported comparison groups.

Data collection of any of the pre-specified NCDs reported in any of the articles was extracted for each cohort study, regardless of the main focus. This included the definition of the disease status, the source of the measurement for diagnosing participants, and the number of time points the data were collected. How the authors reported the NCD (eg, prevalence, incidence, change), the estimate in the total sample, and any associated risk factors reported were also collected. All data extraction was evaluated for correctness and completeness by a second reviewer.

We report studies based on the age group of the population and the type of population. Studies were grouped as ‘children’ or ‘adults’, as reported in the original publications, and ‘mixed’ if there was no age restriction. The type of populations included general, occupational, or clinical. We adapted the WHO STEPwise approach to the NCD risk factor surveillance framework to categorise the essential risk factors (**Table 1**). If the cohort collected data on any of these factors, the definition and source of each of their measurements were extracted.

**Table 1:** Definition of basic, core, or expanded classification within the four domains of risk factor data (adapted from WHO STEPwise [6])

	Demographic & environmental influences	Lifestyle influences	Physiological influences
**Basic**	Age, sex, rural/urban location	Smoking status, alcohol use	Height and weight, waist-circumference, BP, fasting/random blood glucose
**Core**	Education, occupation, socio-economic status	Physical activity, diet	Hip-circumference, lipids profile, urinalysis, infections (HIV, tuberculosis), anaemia
**Expanded**	Indoor/outdoor pollution, occupational exposure	Sexual behaviour, breastfeeding, age at menarche	Birthweight, body composition, oral glucose tolerance test, HbA1c, genetics

BP – blood pressure, HbA1c – glycated hemoglobin

## RESULTS

**Figure 1:**
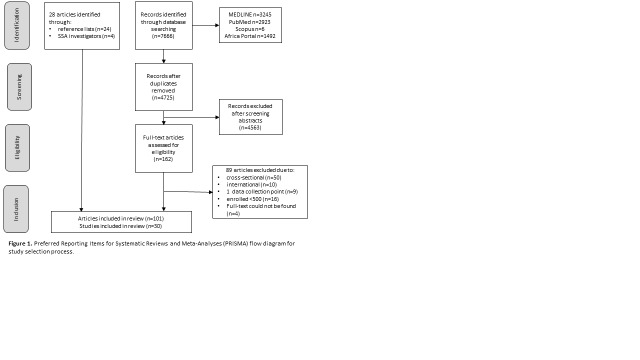
Preferred Reporting Items for Systematic Reviews and Meta-Analyses (PRISMA) flow diagram for study selection process.

The database searches identified 7666 records, of which 2941 were duplicates (**Figure 1**). Through abstract screening, we excluded 4563 records mainly due to publication type (eg, letter), non-SSA setting, cross-sectional data, or non-human participants. A further 89 articles were excluded after assessing the full-text and applying the eligibility criteria (Table S3 in **Online Supplementary Document**). We then added 28 articles from reference lists of included articles, online databases, and SSA study investigators (four out of four investigators who were contacted responded) to give a total of 101 articles included in this review. From these, we identified 30 cohort studies meeting our eligibility criteria; 20 identified through the database search, 6 identified through reference lists, and 2 identified through SSA study investigators.

### Study characteristics

**Figure 2:**
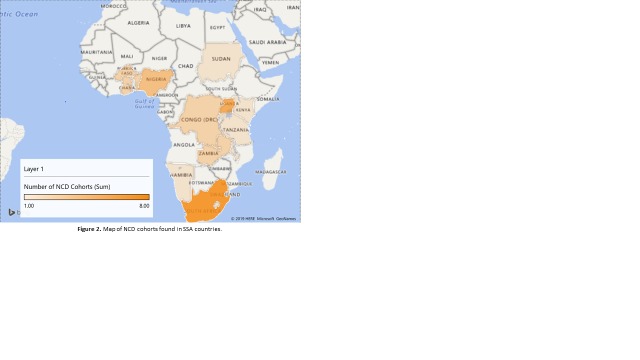
Map of non-communicable disease (NCD) cohorts found in SSA countries.

The study characteristics are reported in **Table 2** and in detail in Tables S4a-d in **Online Supplementary Document**. The majority (27%) of the studies were conducted in South Africa, followed by Uganda (20%), Nigeria (13%), and the Democratic Republic of Congo (7%) (**Figure 2**).

**Table 2:** Characteristics of included studies, including the countries represented, study designs, and duration of each, as well as whether potential to access data was reported and types of stored samples (N = 30)

Characteristics	N (%)
Countries represented*	
Benin	2 (7)
Burkina Faso	2 (7)
Democratic Republic of Congo	2 (7)
Gambia	1 (3)
Ghana	2 (7)
Kenya	1 (3)
Lesotho	1 (3)
Malawi	1 (3)
Mauritius	1 (3)
Namibia	1 (3)
Nigeria	4 (13)
Rwanda	1 (3)
Seychelles	1 (3)
South Africa	8 (27)
Sudan	1 (3)
Tanzania	1 (3)
Uganda	6 (20)
Zambia	2 (7)
**Study duration (years)**	
1-2	8 (27)
3-5	9 (30)
6-10	3 (10)
>10	10 (33)
**Potential to access data?**	
Yes	13 (43)
No	2 (7)
Not reported	15 (50)
**Stored samples:**	
DNA	3 (10)
Blood	1 (3)
Tumour	2 (7)
Serum	1 (3)

*Number of countries is greater than 30 as some studies (N = 3) are set in more than one country, details found in Table S3 in **Online Supplementary Document.**

Three studies were set across multiple SSA countries and seven studies were set in a West African country. The country with the most number of participants enrolled within these studies was South Africa (n = 51 263). Study duration (from recruitment to the end of follow-up) ranged from one year to 38 years with a median of four and a half years (IQR = 2.3-11). Thirteen of the included studies reported the potential to access raw data in a published article while fifteen did not report this and two reported no access. The Birth to Twenty (BT20) study reported the collection and storage of 2200 child samples, along with 2200 samples from one parent. One study reporting cervical cancer data also reported stored cervical samples (both smears and biopsies).

### Participant characteristics

Just over half of the cohorts were in general population (53%) followed by clinical population (47%). There was only one occupational cohort study found, which focused on the health of Basotho gold miners in Lesotho (**Table 3**, Table S5a-d in the **Online Supplementary Document**). Of the general cohort studies, two were birth cohorts – one set in South Africa and the other in Malawi. The number of participants enrolled in each study ranged from 500 participants in the Uganda AIDS Rural Treatment (UARTO) Cohort to over 33 000 participants in the Rakai Community Cohort Study (RCCS) with a median of 2258 participants (IQR = 955-6102) altogether.

**Table 3:** Characteristics of participants within included studies, including sampling frame, number enrolled, age groups reported, sex(s) included, and whether rural/urban location was reported (N = 30)

Characteristics	N(%)
**Sampling frame:**	
General population	16 (53)
Occupational population	1 (3)
Clinical population	13 (43)
**Number enrolled:**	
500-999	9 (30)
1000-4999	11 (37)
5000-10 000	4 (13)
>10 000	6 (20)
**Age groups:**	
Children	3 (10)
Mixed	7 (23)
Adults	20 (67)
**Sex(s) included:**	
Males only	2 (7)
Females only	7 (23)
Mixed	21 (70)
**Rural/urban location:**	
Rural only	8 (27)
Urban only	5 (17)
Mixed	7 (23)
Not reported	10 (33)

The age of participants from all included studies ranged from birth to 95 years. There were three studies including children and young people aged 20 years or less, seven studies including both children and adults, and twenty studies including adults aged 18 years or more. Stratifying on age group, the median number enrolled among the child studies, mixed studies, and adult studies was 779, 5563, and 2045 participants, respectively. The majority (n = 21) of studies included both males and females at a roughly even ratio. Eight studies were set in rural locations, five in urban locations, and seven in mixed rural and urban locations. Rural/urban location was not reported in 10 (33%) of studies.

### NCD data

Hypertension was the most common NCD reported on (n = 23), followed by obesity (n = 16) and diabetes (n = 15) (**Table 4**, Table S6a-d in **Online Supplementary Document**). There were very few studies collecting data on COPD and we did not find any studies collecting data on asthma that met our eligibility criteria. Out of the pre-specified cancers, we only found studies reporting on cervical (n = 3) and breast (n = 1) cancers that met the criteria. Half of the studies (50%) reported the collection of three or more chronic NCDs. The majority of studies included data on hypertension, as well as diabetes and obesity. There were four studies that collected data on only biomarkers for CKD. Two studies, the Africa Wits-INDEPTH partnership for Genomic (AWI-Gen) Studies (with more than 10 000 participants) and the African Breast Cancer Disparities in Outcomes (ABC-DO) Study (with nearly 2000 participants), collected data on five of the six NCDs. In general, the NCD data collected by these studies were objectively measured for the purpose of the study.

**Table 4:** NCD data characteristics including total number of studies reporting each and percentage of each NCD reported in terms of prevalence, incidence, change in mean, or not reported (N = 30)

Characteristics	N (%)
NCDs*	
Hypertension	23 (77)
Diabetes	15 (50)
Obesity	18 (60)
CKD	6 (20)
COPD	2 (7)
Breast cancer	1 (3)
Cervical cancer	3 (10)
Prostate cancer	0 (0)
Oesophageal cancer	0 (0)
**Number of NCDs studied in each:**	
1	12 (40)
2	3 (10)
≥3	15 (50)
**Reporting of NCD:**	
Aetiology (prevalence)	19 (63)
Incidence	3 (10)
Prognosis (change)	4 (13)
Not reported	4 (13)

NCD – non-communicable diseases

*Number of NCDs reported on is greater than 30 as some studies collected data on more than one NCD.

### Risk factor data

**Figure 3:**
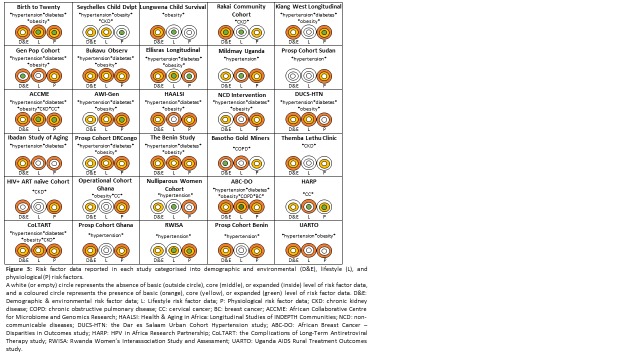
Risk factor data reported in each study categorised into demographic and environmental (**D&E**), lifestyle (**L**), and physiological (**P**) risk factors. A white (or empty) circle represents the absence of basic (outside circle), core (middle), or expanded (inside) level of risk factor data, and a coloured circle represents the presence of basic (orange), core (yellow), or expanded (green) level of risk factor data. D&E – Demographic & environmental risk factor data; L – Lifestyle risk factor data; P – Physiological risk factor data; CKD – chronic kidney disease; COPD – chronic obstructive pulmonary disease; CC – cervical cancer; BC – breast cancer; ACCME – African Collaborative Centre for Microbiome and Genomics Research; HAALSI – Health & Aging in Africa – Longitudinal Studies of INDEPTH Communities; NCD – non-communicable diseases; DUCS-HTN – the Dar es Salaam Urban Cohort Hypertension study; ABC-DO – African Breast Cancer – Disparities in Outcomes study; HARP – HPV in Africa Research Partnership; CoLTART – the Complications of Long-Term Antiretroviral Therapy study; RWISA – Rwanda Women’s Interassociation Study and Assessment; UARTO – Uganda AIDS Rural Treatment Outcomes study.

The characteristics of risk factor data collected by included studies varied greatly (**Figure 3**) and are presented in greater detail in Appendix Table 6a-e. The majority (n = 22) of studies reported the collection of data on at least one demographic/environmental, lifestyle, or physiological risk factor. There were two studies, the Birth to Twenty cohort (BT20) and the ACCME’s HPV and Cervical Cancer Study, which reported the collection of almost all the pre-specified basic, core, and expanded risk factors in each category. One study, the prospective cohort in Sudan, only reported physiological risk factor data; it did not include data such as rural/urban location or smoking status. Environmental data were collected by only three studies – both the General Population Cohort (GPC) and the RCCS reported data on aflatoxin exposure and the Basotho Gold Miners Cohort reported data on silica dust exposure.

### Methodological characteristics

Out of all the included studies, 37% (n = 11) utilised random sampling of participants, 53% (n = 16) utilised a convenience sample and 10% (n = 3) did not report sampling method. There was a total of 11 studies that enrolled more than 80% of eligible participants (2 child studies, 3 mixed studies, and 6 adult studies), while 10 studies were unclear or did not report the number of participants who were eligible to participate. 43% (n = 13) of the included studies were able to follow-up at least 70% of their enrolled participants, 40% (n = 12) were unclear or did not report the number of participants followed up, and two studies (AWI-Gen and HAALSI) had not yet completed its first round of follow-up. The majority (87%) of studies – all 3 child studies, all 7 mixed studies, and 16 adult studies - collected NCD data objectively for the purpose of the study. These studies also reported either using validated or standardised methods (n = 11), checking their methods against a ‘gold standard’ (n = 2), or calibrating equipment (n = 4). Demographic and lifestyle risk factor data was self-reported in these studies, however, many used standardised questionnaires such as the WHO STEPwise questionnaire, making the data comparable to other studies. Physiological risk factor data was objectively measured in 63% of included studies, obtained from medical records in 7%, and not reported in 10%.

## DISCUSSION

### Main findings

Our comprehensive search strategy identified 30 studies (reported in 101 papers) prospectively collecting longitudinal data on NCDs and their risk/prognostic factors within SSA. Eighteen countries, out of the fifty countries in SSA, were represented among the included studies, in the West, East, Central and Southern Africa with the majority set in South Africa. The total number of participants under study was highest in South Africa, followed by Uganda and Zambia. There were three studies that reported including only children and young people under the age of 20 years and seven studies that reported including both children and adults.

Regardless of their main focus, the majority of studies collected data on a combination of hypertension, diabetes, and obesity. These studies also collected robust risk factor data. Of the six studies that reported the collection of CKD data, half reported collecting additional data on at least hypertension; a closely associated risk factor. COPD is the third leading cause of death worldwide, and little is known about the prevalence in SSA [14]. The two clinical studies collecting COPD data reported few risk factor data. Many cross-sectional studies with asthma data were found during the selection processes, but we did not find one cohort study meeting the eligibility criteria. Breast and cervical cancers are the two leading causes of cancer mortality among women in SSA, with shifts in risk factors often providing the only indications for prevention programmes [15]. Three studies reported breast or cervical cancer data and included limited risk factor data. The burden of prostate and oesophageal cancers in SSA is also unclear, reflected by the lack of data found.

Less than half of the included studies enrolled more than 80% of eligible participants or were able to follow-up more than 70% of enrolled participants. This highlights some methodological challenges faced when conducting these studies that should be considered for future studies. We found that NCD data and risk factor data were objectively measured for the purpose of the study in the majority of the included studies. Most of this data was measured using validated measures or standard procedures.

The main strengths of our review are the wide scope of diseases and the inclusion of studies with large numbers of participants. Similar reviews of existing cohorts in SSA have been carried out but were limited in scope. One study was a systematic review of birth cohorts, including only participants at less than 10 years of age at enrolment [9]. Another two studies included cohorts with very few participants [10,16]. Unlike these reviews, ours is a comprehensive review of the cohort studies from articles published after the year 2000 covering all ages, four different regions and a wide range of NCDs, providing a useful resource for assessing the available evidence in SSA.

Although we used a systematic approach to search for eligible studies and did not limit our eligibility by any specific funder, it is possible that our review is at risk of publication bias. There may be cohorts of participants under study in these populations collecting data on NCDs that we were not able to capture because the data has not been published or has been published in grey literature, such as PhD theses. In addition, almost all of the published articles found were in English, with a few in French or Portuguese. It is likely that more non-English articles exist but were not found in the database searches because they lack international readership. To overcome this, we specifically contacted study investigators from Francophone West Africa in order to reduce publication bias. As any review of published studies our study might have been affected by reporting bias because it was only possible to extract data that has been published, but some studies might have not reported all the relevant information.

## CONCLUSION

Scoping reviews are a useful tool to map existing data available and to identify any gaps in research. In this scoping review, we presented the existing data on six NCDs, and their risk factors, which has been collected by large cohort studies in SSA. In terms of the total number of studies, regardless of their main focus, most of the existing data available were on hypertension, followed by diabetes and obesity, which is probably due to the relative ease of data collection and possibly a higher prevalence of these conditions. In terms of the total number of participants reporting biomarkers, most of the existing data available were on biomarkers of CKD. Very few data were available for COPD, and the studies collecting this data utilised self-report or invalid methods. Existing data was available for breast and cervical cancers but not for prostate or oesophageal. Risk factor data available varied widely and was not always clearly-reported.

### Implications

To the best of our knowledge this is the first time NCD cohorts in SSA have been mapped out, providing key visibility to researchers, both locally and internationally, to identify areas for collaboration and further interrogation. This is especially important when Africa is experiencing both a demographic transition with an ageing population and an epidemiological transition with an increased number of NCD. Our findings showed that currently most cohorts are focused on a specific NCD with few addressing a combination of NCDs, or their shared risk factors. We hope that the finding that multi-morbidity is not properly included in this type of cohorts will call the attention of interested researchers and new cohorts of NCD will be designed to include this increasingly phenomenon in adults and older population. There is also a lack of standardized methodology and important limitations regarding sampling and follow up of participants, which may require clear and standardised international guidelines. Furthermore, only half of the cohorts were in the general population. This is particularly relevant for SSA where, compared to other regions, people have lower accessibility to health services and many people are not aware of their chronic conditions. In order to better understand the epidemiology of NCDs, including aspects related to aetiology, burden and prognosis, we need large population studies across different countries and demographic groups, with adequate sampling, long-term follow-up, detailed phenotyping and standardised assessments of NCDs and their risk factors.

One exemplar of this type of initiative is the recently funded African NCD Longitudinal Data Alliance (ANDLA) which brings together data currently from eight population-based cohorts from five countries across sub-Saharan Africa to create large, harmonised data sets that are robust and powerful enough to address important research questions with generalizable findings that partners could not address individually. ANDLA will provide a dynamic and sustainable platform of population-based data collected over time (longitudinal-data). The data will be standardised and made available in an easily usable form and will focus on NCD and their risk factors. It will be a unique large-scale data resource for understanding NCD in Africa to inform and shape effective policy. Our review can also be useful to flag important knowledge gaps that need addressing by initiatives such as ANDLA.

The key to the control of the NCD epidemic in SSA is primary prevention and prognosis, the basis of which is surveillance and involves the systematic collection of high quality data [6]. This information is essential for advising future research, as well as public health policies. There are many studies collecting high quality data that could easily be built upon to include the full range of NCDs. These studies could be useful to study the life-course approach to NCDs and their interaction. In addition, more than half of the included studies were sampled from the general population, which can be useful for designing local policies. The large Network of Health and Demographic Surveillance System sites (HDSS) distributed along SSA, provides a longitudinal demographic platform where researchers can design large population-based studies for chronic conditions in a well stablished research environment. This is the case, for example of the Health and Aging in Africa: a longitudinal study of an INDEPTH community (HAALSI).

Highlighting the importance of the existing cohorts could be used by researchers to leverage additional funding from local policy makers to extend, expand, or enrich their current research platforms. For policy makers this manuscript showcases the cohorts that could be used to identify locally relevant outcomes which in turn could shape, or target, future interventions. For policy makers this manuscript also provides clarity on existing cohort gaps, providing an opportunity for funding to be directed in the most impactful direction.

Groups like the Coalition for African Research Innovation (CARI) [17] have exposed the bias in SSA research funding towards the infectious disease space. Given the current limitations in funding available for NCD research in SSA, mapping out and understanding infectious disease cohorts that could lend themselves to study NCDs or their interaction, would be a logical future step to complement the current report. This is particularly important given the emergence of ID/NCD dual disease burden in LMICs. Knowledge of the existing NCD cohort environment would enable both funders and researchers to readdress this bias.

Regarding some of the methodological challenges, future studies should consider design strategies, like using health tools and wearables that could facilitate consistency in data collection to ensure consistent and comparable data. Also, better communication and coordination between the different research groups could be beneficial to explore potential collaborations to maximize the impact of each study improve our global understanding of chronic NCDs, and their risk factors, in SSA.

## Additional material

Online Supplementary Document

## References

[1] Sitas F, Parkin M, Chirenje Z, Stein L, Mqoqi N, Wabinga H. Disease and Mortality in Sub-Saharan Africa. Washinton DC: The International Bank for Reconstruction and Development/The World Bank. 2006. 21290641

[2] Nyirenda MJ Non-communicable diseases in sub-Saharan Africa: Understanding the drivers of the epidemic to inform intervention strategies. Int Health. 2016;8:157-8. 10.1093/inthealth/ihw021 27178673

[3] Holmes MD Dalal S Volmink J Adebamowo CA Njelekela M Fawzi WW Non-communicable diseases in sub-Saharan Africa: the case for cohort studies. PLoS Med. 2010;7:e1000244. 10.1371/journal.pmed.1000244 20485489 PMC2867939

[4] Petersen M Yiannoutsos CT Justice A Egger M Observational Research on NCDs in HIV-positive populations: Conceptual and methodological considerations. J Acquir Immune Defic Syndr. 2014;67:S8-16. 10.1097/QAI.0000000000000253 25117964 PMC4317266

[5] Khaledifar A Hashemzadeh M Solati K Poustchi H Bollati V Ahmadi A The protocol of a population-based prospective cohort study in southwest of Iran to analyze common non-communicable diseases: Shahrekord cohort study. BMC Public Health. 2018;18:660. 10.1186/s12889-018-5364-2 29801446 PMC5970455

[6] World Health Organization. The WHO STEPwise approach to Surveillance of noncommunicable diseases. Geneva: WHO; 2003.

[7] Peters MDJ Godfrey CM Khalil H McInerney P Parker D Soares CB Guidance for conducting systematic scoping reviews. Int J Evid-Based Healthc. 2015;13:141-6. 10.1097/XEB.0000000000000050 26134548

[8] Kengne AP Ntyintyane LM Mayosi BM A systematic overview of prospective cohort studies of cardiovascular disease in sub-Saharan Africa. Cardiovasc J Afr. 2012;23:103-12. 10.5830/CVJA-2011-042 21901226 PMC3734756

[9] Campbell A Rudan I Systematic review of birth cohort studies in Africa. J Glob Health. 2011;1:46-58. 23198102 PMC3484737

[10] Taieb F Madec Y Cournil A Delaporte E. Virological success after 12 and 24 months of antiretroviral therapy in sub-Saharan Africa: Comparing results of trials, cohorts and cross- sectional studies using a systematic review and meta-analysis. PLoS One. 2017;12:e0174767. 10.1371/journal.pone.0174767 28426819 PMC5398519

[11] Defor S Kwamie A Agyepong IA Understanding the state of health policy and systems research in West Africa and capacity strengthening needs: scoping of peer-reviewed publications trends and patterns 1990–2015. Health Res Policy Syst. 2017;15:55. 10.1186/s12961-017-0215-7 28722555 PMC5516843

[12] INDEPTH Network. Available: http://www.indepth-network.org/. Accessed: 13 December 2017.

[13] Reviewers’ Manual: Methodology for JBI Scoping Reviews. 2015.

[14] Salvi S The silent epidemic of COPD in Africa. Lancet Glob Health. 2015;3:e6-7. 10.1016/S2214-109X(14)70359-6 25539971

[15] Tsu VD Jeronimo J Anderson BO Why the time is right to tackle breast and cervical cancer in low-resource settings. Bull World Health Organ. 2013;91:683-90. 10.2471/BLT.12.116020 24101784 PMC3790214

[16] Kengne AP Ntyintyane LM Mayosi BM A systematic overview of prospective cohort studies of cardiovascular disease in sub-Saharan Africa. Cardiovasc J Afr. 2012;23:103-12. 10.5830/CVJA-2011-042 21901226 PMC3734756

[17] AESA. Coalition for African Research and Innovation (CARI): The need for robust African research & development. 2019. https://aasciences.ac.ke/cari. Accessed: 19 March 2019.

