# Supplementary material for: Non-communicable diseases in sub-Saharan Africa: a scoping review of large cohort studies
Source: J Glob Health. 2019 Aug 6;9(2):020409. doi: 10.7189/jogh.09.020409 (PMC6684871; doi:10.7189/jogh.09.020409)
Supplement: Online Supplementary Document [file jogh-09-020409-s001.pdf]

J Glob Health 2019; 9: 020409

SUPPLEMENTAL INFORMATION

**Supplemental Table**	**Page**
STable 1: MEDLINE search strategy	2
STable 2: Data extraction form	6
STable 3: Studies that were excluded after obtaining the full-text article	8
STable 4a: Characteristics of included studies with only children and young adults aged <20 years, sampled from general populations	32
STable 4b: Characteristics of included studies with mixed (children and adults) populations, sampled from general and from clinical populations	33
STable 4c: Characteristics of included studies with only adults aged >18 yrs, sampled from general populations	35
STable 4d: Characteristics of included studies with only adults aged >18yrs, sampled from occupational and clinical populations	37
STable 5a: Participant characteristics of included studies with children and young adults aged <20 years, sampled from general populations	40
STable 5b: Participant characteristics of included studies with mixed (children and adults) populations, sampled from general and clinical populations	41
STable 5c: Participant characteristics of included studies with only adults aged >18 yrs, sampled from general populations	43
STable 5d: Participant characteristics of included studies with only adults aged >18 yrs, sampled from occupational and clinical populations	45
STable 6a: Characteristics of chronic NCD data of included studies with children and young adults aged <20 years, sampled from general populations	48
STable 6b: Characteristics of chronic NCD data of included studies with mixed (children and adults) populations, sampled from general and clinical populations	50
STable 6c: Characteristics of chronic NCD data of included studies with adults aged >18 years, sampled from general populations	54
STable 6d: Characteristics of chronic NCD data of included studies with adults aged >18 years, sampled from occupational and clinical populations	59
STable 7a: Characteristics of risk factor data of included studies with children and young adults aged <20 years, sampled from general populations	69
STable 7b: Characteristics of risk factor data of included studies with mixed (children and adults) populations, sampled from general and clinical populations	72
STable 7c: Characteristics of risk factor data of included studies with adults aged >18 years, sampled from general populations	80
STable 7d: Characteristics of risk factor data of included studies with adults aged >18 years, sampled from occupational and clinical populations	89

STable 1: MEDLINE search strategy

#	Search terms	Results
1	"africa south of the sahara"/ or africa, central/ or africa, eastern/ or africa, southern/ or africa, western/	18735
2	("africa south of the sahara" or sub-saharan africa or central africa or eastern africa or southern africa or western africa).ti,ab.	20044
3	Benin/	1339
4	(Benin or Dahomey).ti,ab.	2561
5	Burkina Faso/	2824
6	(Burkina Faso or Burkina Fasso or Upper Volta).ti,ab.	3149
7	Burundi/	603
8	Burundi.ti,ab.	633
9	Central African Republic/	721
10	(Central African Republic or Ubangi-Shari).ti,ab.	817
11	Chad/	653
12	Chad.ti,ab.	896
13	Comoros/	262
14	(Comoros or Comoro Islands or Mayotte or Iles Comores).ti,ab.	438
15	"Democratic Republic of the Congo"/	3662
16	((demogratic republic adj2 congo) or belgian congo or zaire).ti,ab.	3432
17	Eritrea/	272
18	Eritrea.ti,ab.	362
19	Ethopia/	9799
20	Ethiopia.ti,ab.	8585
21	Gambia/	2295
22	Gambia.ti,ab.	1950
23	Guinea/	903
24	(Guinea not (New Guinea or Guinea Pig* or Guinea Fowl)).ti,ab.	3086
25	Guinea-Bissau/	853
26	(Guinea-Bissau or Portuguese Guinea).ti,ab.	845
27	Liberia/	1053
28	Liberia.ti,ab.	1140
29	Madagascar/	3045
30	(Madagascar or Malagasy Republic).ti,ab.	3619
31	Malawi/	4369
32	(Malawi or Nyasaland).ti,ab.	4703
33	Mali/	2107
34	Mali.ti,ab.	2652
35	Mozambique/	1989
36	(Mozambique or Mocambique or Portuguese East Africa).ti,ab.	2509
37	Niger/	1064
38	(Niger not (Aspergillus or Peptococcus or Schizothorax or Cruciferae or Gobius or Lasius or Agelastes or Melanosuchus or radish or Parastromateus or Orius or Apergiullus or Parastromateus or Stomoxys)).ti,ab.	2491
39	Rwanda/	1998
40	(Rwanda or Ruanda).ti,ab.	2039
41	Senegal/	5297
42	senegal.ti,ab.	4607
43	Sierra Leone/	1274
44	Sierra Leone.mp.	1764
45	Somalia/	1415
46	Somalia.ti,ab.	1041
47	South Sudan/	88
48	south sudan.ti,ab.	315
49	Tanzania/	9821
50	(Tanzania or Tanganyika or Zanzibar).ti,ab.	9773
51	Togo/	1000
52	(Togo or Togolese Republic).ti,ab.	1117
53	Uganda/	10311
54	Uganda.ti,ab.	9919

55	Zimbabwe/	5354
56	(Zimbabe or Rhodesia).ti,ab.	4775
57	Angola/	867
58	angola.ti,ab.	1034
59	Cameroon/	4740
60	Cameroon.ti,ab.	4947
61	Cape Verde/	153
62	(Cape Verde or Cabo Verde).ti,ab.	443
63	Congo/	1666
64	(congo not ((democratic republic adj3 congo) or congo red or crimean-congo)).ti,ab.	2090
65	Cote d'Ivoire/	2864
66	(Cote d'Ivoire or Ivory Coast).ti,ab.	3079
67	Ghana/	6561
68	(Ghana or Gold Coast).ti,ab.	6694
69	Kenya/	13981
70	kenya.mp.	16901
71	Lesotho/	367
72	(Lesotho or Basutoland).ti,ab.	510
73	Mauritania/	398
74	Mauritania.ti,ab.	478
75	Nigeria/	25460
76	Nigeria.ti,ab.	19666
77	Atlantic Islands/	724
78	(sao tome adj2 principe).ti,ab.	106
79	Sudan/	4378
80	(Sudan not south sudan).ti,ab.	5749
81	Swaziland/	482
82	Swaziland.ti,ab.	634
83	Zambia/	3916
84	(Zambia or Northern Rhodesia).ti,ab.	3808
85	Botswana/	1525
86	(Botswana or Bechuanaland or Kalahari).ti,ab.	1788
87	Equatorial Guinea/	230
88	(Equatorial Guinea or Spanish Guinea).ti,ab.	336
89	Gabon/	1360
90	(Gabon or Gabonese Republic).ti,ab.	1414
91	Mauritius/	513
92	(Mauritius or Agalega Islands).ti,ab.	725
93	Namibia/	905
94	Namibia.ti,ab.	1038
95	South Africa/	37465
96	South Africa.ti,ab.	23379
97	Seychelles/	328
98	Seychelles.ti,ab.	541
99	or/1-98 [ALL COUNTRIES IN SUB-SAHARAN AFRICA]	216458
100	Epidemiologic studies/	7708
101	exp Cohort studies/	1749070
102	100 or 101	1755715
103	(cohort stud*).ti,ab.	131703
104	(cohort analy*).ti,ab.	5335
105	(follow-up).ti,ab.	736920
106	(followup).ti,ab.	17427
107	(prospective stud*).ti,ab.	137524
108	(longitudinal stud*).ti,ab.	55691
109	(retrospective stud*).ti,ab.	108903
110	(panel stud*).ti,ab.	1616
111	(panel analy*).ti,ab.	382
112	103 or 104 or 105 or 106 or 107 or 108 or 109 or 110 or 111 or 112	1088161
113	102 or 112	2154971

114	exp Renal Insufficiency, chronic/	102826
115	((chronic or acute or reduced or progress*) adj6 (renal or kidney) adj5 (failure* or disease* or insufficienc* or injur* or damage* or impairment* or function* or dysfunction*)).ti,ab.	112000
116	((end-stage* or endstage*) adj4 (renal or kidney)).ti,ab.	32416
117	(ESRD or ESKD or ESKF or CKD or CRD or CKF).ti,ab.	31609
118	(proteinuri* or creatinine*).ti,ab.	115964
119	(glomerul* adj3 filtrat* adj3 rate*).ti,ab.	34048
120	(GFR or eGFR).ti,ab.	53925
121	114 or 115 or 116 or 117 or 118 or 119 or 120	317540
122	Lung Diseases, Obstructive/	18090
123	exp Pulmonary Disease, Chronic Obstructive/	48427
124	exp Asthma/	119532
125	122 or 123 or 124	178292
126	(obstruct* adj3 (pulmonary or lung* or airway* or bronch* or respirat*)).ti,ab.	66118
127	(COPD or COAD or COBD or AECB).ti,ab.	33189
128	(asthma* or wheez* or emphy#ema or dyspnea).ti,ab.	178358
129	(chronic* adj3 (pulmonary or lung* or airway* or bronch* or respirat*)).ti,ab.	78568
130	(spirometry).ti,ab.	12714
131	126 or 127 or 128 or 129 or 130	269986
132	125 or 131	299822
133	Cardiovascular diseases/	130657
134	exp Hypertension/	239229
135	133 or 134 or 134	505365
136	hypertens*.ti,ab.	354324
137	((elevat* or high or increase*) adj3 blood adj pressur*).ti,ab.	35234
138	((systolic or diastolic or arter*) adj3 pressur*).ti,ab.	173368
139	(SBP or DBP).ti,ab.	20660
140	136 or 137 or 138 or 139	1543276
141	135 or 140	1688081
142	Neoplasms/	385438
143	Prostate/	33031
144	exp Prostatic Neoplasms/	114402
145	Uterine Cervical Neoplasms/	69196
146	exp Breast Neoplasms/	263917
147	Carcinoma, Ductal, Breast/	14556
148	Esophageal Neoplasms/	45865
149	exp Liver Neoplasms/	150300
150	142 or 143 or 144 or 145 or 146 or 147 or 148 or 149	1008626
151	(cancer* or neoplasm* or carcinoma* or tumo?r* or metasta* or malig* or adenocarcinoma* or sarcoma* or leiomyosarcoma* or dcis or duct* or intraduct* or medullary or tubular).ti,ab.	2789143
152	(prostat* or cerv* or breast or mammar* or esohag* or oesaphag* or liver*).ti,ab.	1401244
153	151 and 153	639282
154	150 or 153	1187973
155	exp Diabetes Mellitus, Type 2/	114375
156	exp Hyperglycemia/	32712
157	exp Hyperinsulinism/	78640
158	155 or 156 or 157	199505
159	(Type* adj3 ("2" or II or two*) adj3 (diabete* or diabetic*)).ti,ab.	102566
160	((Non-insulin* or Non insulin* or Noninsulin*) adj3 depend* adj3 (diabete* or diabetic*)).ti,ab.	11397
161	(NIDDM).ti,ab.	6717
162	(hyperglyc?emi* or hyperinsulin*).ti,ab.	65353
163	159 or 160 or 161 or 162	165208
164	158 or 163	249545
165	exp Obesity/	186186
166	exp Overweight/	191324
167	165 or 166	194030

168	(obesity or obese).ti,ab.	217631
169	(overweight or over-weight or overweight).ti,ab.	49565
170	(adipos*).ti,ab.	79728
171	168 or 169 or 170	283311
172	167 or 171	324315
173	121 or 132 or 141 or 154 or 164 or 170	3674613
174	99 and 113 and 173	4123
175	limit 175 to (humans and yr="2000-Current")	3245

STable 2: Data extraction form

**Cohort Name**	
Study ID of relevant publication(s)	
Corresponding authors' contact details	
**Study Characteristics**	
Country	
Study design	
Start date	
End date	
Total duration of study	
# of data collection points	
Main focus	
Funding of study	
Potential for data access?	
**Participant Characteristics**	
Sampling frame	
Method of sampling	
Inclusion/Exclusion criteria	
# eligible	
# enrolled	
Sample size at final evaluation	
Response rate	
Age (mean)	
Possible to stratify on age?	
Sex (%)	
Ethnicity (%)	
Rural/urban (%)	
Comparison groups reported (N)	
**Chronic NCD data**	
Chronic NCD(s)<br>(repeat for each NCD)	
Chronic NCD	
Definition	
Source of measurement	
Time points measured	
Reporting of chronic NCD	
Estimate in total sample	
Associated risk factor(s) reported	
**Risk Factor data**	
Demographic & Environmental risk factors<br>(ie education, SES, occupation, indoor/outdoor pollution, occupational exposure; repeat for each risk factor)	
Risk factor	
Definition	
Time points measured	
Source of measurement	
Lifestyle risk factors<br>(ie smoking status, alcohol use, physical activity, diet, sexual behaviour, breastfeeding, age at menarche; repeat for each risk factor)	
Risk factor	

Definition	
Time points measured	
Source of measurement	
Physiological risk factors<br>(ie BP, height, weight, WC, fasting blood glucose, HC, lipids, urinalysis, HIV infection, TB infection, anaemia, body composition, OGTT, genetics; repeat for each risk factor)	
Risk factor	
Definition	
Time points measured	
Source of measurement	
Stored samples?<br>(repeat for each type of sample)	
Type of sample	
Method of storage	
**Results**	
Loss to follow-up<br>(if yes, give reasons)	
**Notes**	

STable 3: Studies that were excluded after obtaining the full-text article

Study ID	Identified through	Title	Journal	Country	Study Design	# of participants	# of data collection points	follow-up time	Reason for exclusion
Abdissa 2014	database	Spectrum of cardiovascular diseases among Ethiopian patients in Tikur Anbessa Specialised University Teaching Hospital, Addis Abba.	Ethiopian medical journal	Ethiopia	cohort study	<500			small
Accorsi 2009	database	Poverty, inequality and health: the challenge of the double burden of disease in a non-profit hospital in rural Ethiopia.	Transactions of the Royal Society of Tropical Medicine and Hygiene	Ethiopia	cross-sectional				cross-sectional
Amberbir 2011	database	Effects of helicobacter pylori, geohelminth infection and selected commensal bacteria on the risk of allergic disease and sensitization in 3-year-old Ethopian children.	Clinical and experimental allergy	Ethiopia	cross-sectional				cross-sectional

Study ID	Identified through	Title	Journal	Country	Study Design	# of participants	# of data collection points	follow-up time	Reason for exclusion
Amberbir 2011c	database	Frequent use of paracetamol and risk of allergic disease among women in an Ethiopian population.	PloS one	Ethiopia	cross-sectional				cross-sectional
Anastos 2010	database	Risk factors for cervical precancer and cancer in HIV-infected, HPV-positive Rwandan women.	PloS one	Rwanda	cross-sectional				cross-sectional
Arokiasamy 2017	database	Chronic noncommunicable diseases in 6 low- and middle-income countries: findings from wave 1 of the world health organization's study on global ageing and adult health (SAGE).	American journal of epidemiology	China, Ghana, India, Mexico, Russia, South Africa	cross-sectional				cross-sectional
Arokiasamy 2017	database	Chronic noncommunicable diseases in 6 low- and middle-income countries: findings from wave 1 of the	American journal of epidemiology	China, Ghana, India, Mexico, Russia, South Africa	international study				international

Study ID	Identified through	Title	Journal	Country	Study Design	# of participants	# of data collection points	follow-up time	Reason for exclusion
		world health organization's study on global ageing and adult health (SAGE).							
Atiase 2015	database	A comparison of indices of glucose metabolism in five black populations: data form medling the epidemiologic transition study.	BMC public health	US, Jamaica, Ghana, South Africa, Seychelles	cross-sectional				cross-sectional
Atiase 2015	database	A comparison of indices of glucose metabolism in five black populations: data form medling the epidemiologic transition study.	BMC public health	US, Jamaica, Ghana, South Africa, Seychelles	cross-sectional				cross-sectional
Basu 2013	database	Social epidemiology of hypertension in middle-income countries: determinants of prevalence, diagnosis, treatment, and control in the WHO SAGE study.	Hypertension	China, Ghana, India, Mexico, Russia, South Africa	cross-sectional				cross-sectional

Study ID	Identified through	Title	Journal	Country	Study Design	# of participants	# of data collection points	follow-up time	Reason for exclusion
Beguy 2015	database	Health & Demographic Surveillance System Profile: The Nairobi Urban Health and Demographic Surveillance System (NUHDSS)	Int J Epidemiol	Kenya	cross-sectional				cross-sectional
Belyhun 2010	database	Prevalence and risk factors of wheeze and eczema in 1-year-old children: the Butajira birth cohort, Ethiopia.	Clinical and experimental allergy	Ethiopia	cross-sectional				cross-sectional
Biccard 2012	database	What is the best pre-operative risk stratification tool for major adverse cardiac events following elective vascular surgery? A prospective observational cohort study evaluation pre-operative myocardial ischaemia monitoring and biomarker anlaysis.	Anaesthesia	South Africa	cross-sectional				cross-sectional

Study ID	Identified through	Title	Journal	Country	Study Design	# of participants	# of data collection points	follow-up time	Reason for exclusion
Biccard 2012	database	What is the best pre-operative risk stratification tool for major adverse cardiac events following elective vascular surgery? A prospective observational cohort study evaluation pre-operative myocardial ischaemia monitoring and biomarker analysis.	Anaesthesia	South Africa	cross-sectional analysis of cohort data				cross-sectional
Botha 2014	database	Associations of suPAR with lifestyle and cardiometabolic risk factors.	European journal of clinical investigation	South Africa	cross-sectional				cross-sectional
Botha 2015	database	Soluble urokinase plasminogen activator receptor as prognostic marker of all-cause and cardiovascular mortality in a black population.	International journal of cardiology	South Africa	cross-sectional				cross-sectional

Study ID	Identified through	Title	Journal	Country	Study Design	# of participants	# of data collection points	follow-up time	Reason for exclusion
Carrillo-Larco 2014	database	Short sleep duration and childhood obesity: cross-sectional analysis in Peru and patterns in four developing countries	PloS one	Ethiopia, India, Peru, Vietnam	cross-sectional analysis of cohort data	<500			small
Chalumeau 2002	database	Can clinical risk factors for late stillbirth in West Africa be detected during antenatal care or only during labour?	International journal of epidemiology	Ivory Coast, Mali, Senegal, Niger, Mauritania, Burkina Faso, Senegal	cross-sectional				cross-sectional
Chiolero 2011	database	Birth weight, weight change, and blood pressure during childhood and adolescence: a school-based multiple cohort study	J Hypertens	Seychelles	cross-sectional				cross-sectional
Chirenda 2000	database	Association of HIV infection with the development of severe and complicated malaria cases at a	Cent Afr J Med	Zimbabwe	Prospective cohort	<500			small

Study ID	Identified through	Title	Journal	Country	Study Design	# of participants	# of data collection points	follow-up time	Reason for exclusion
		rural hospital in Zimbabwe							
Chirwa 2014	database	Postnatal growth velocity and overweight in early adolescents: a comparison of rural and urban African boys and girls.	Americna journal of human biology	South Africa, Malawi	child cohorts	<500			small
Chokunonga 2011	database	Cancer survival in Harare, Zimbabe, 1993-1997.	IARC scientific publications	Zimbabwe	follow-up	<500			small
Clark 2015	database	Cardiometabolic disease risk and HIV status in rural South Africa: establishing a baseline	BMC public health	South Africa	cross-sectional				cross-sectional
Cois 2014	database	Analysing the socioeconomic determinants of hypertension in South Africa: a structural equation modelling approach.	BMC public health	South Africa	cross-sectional				cross-sectional
Cooper 2015	database	Elevated hypertension risk for African-origin populations in	Journal of hypertension	Ghana, South Africa,	cross-sectional				cross-sectional

Study ID	Identified through	Title	Journal	Country	Study Design	# of participants	# of data collection points	follow-up time	Reason for exclusion
		biracial societies: modeling the Epidemiologic Transition Study.		Seychelles					
Crampin 2012	database	Profile: the Karonga Health and Demographic Surveillance System	Int J Epidemiol	Malawi	cross-sectional				cross-sectional
Damorou 2008	database	Cardiovascular emergencies and their morbimortality at the hospital. Report of 733 cases at the CHU campus in Lome (national reference hospital in Togo).	Mali medicine	Togo	cross-sectional				cross-sectional
Davey 2005	database	Wheeze, allergic sensitization and geohelminth infection in Butajira, Ethiopia.	Clinical and experimental allergy	Ethiopia	cross-sectional				cross-sectional
Dawam 2000	database	Benign prostatic hyperplasia and prostate carcinoma in native Africans.	BJU international	Nigeria	cross-sectional				cross-sectional
Deckert 2017	database	Assessment of renal function in routine care of	PloS one	Zambia	cross-sectional				cross-sectional

Study ID	Identified through	Title	Journal	Country	Study Design	# of participants	# of data collection points	follow-up time	Reason for exclusion
		people living with HIV on ART in a resource-limited setting in urban Zambia.							
Deckert 2017	database	Assessment of renal function in routine care of people living with HIV on ART in a resource-limited setting in urban Zambia.	PloS one	Zambia	cohort	>500	1		1 data collection point
Deeg 2008	database	A comparison of cardiovascular disease risk factor biomarkers in African Americans and Yoruba Nigerians	Ethnicity & disease	USA, Nigeria					international
Denslow 2011	database	Bacterial vaginosis as a risk factor for high-grade cervical lesions and cancer in HIV-seropositive women.	International journal of gynaecology and obstetrics	South Africa	cross-sectional				cross-sectional
Dolo 2003	database	Epidemiology of malaria in a village of Sudanese savannah in Mali		Mali	cross-sectional				cross-sectional

Study ID	Identified through	Title	Journal	Country	Study Design	# of participants	# of data collection points	follow-up time	Reason for exclusion
		(Bancoumana). Anti-TRAP and anti-CS humoral immunity response.							
Dorie 2015	database	Screening of cerebral vasculopathy in sickle cell anemia children using transcranial Doppler.		Mali	cross-sectional				cross-sectional
Dugas 2014	database	Comparisons of intensity-duration patterns of physical activity in the US, Jamaica and 3 African countries.	BMC public health	Ghana, South Africa, Seychelles, Jamaica, USA					international
Duong 2013	database	Global differences in lung function by region (PURE): an international, community-based prospective study.	The Lancet. Respiratory medicine	17 countries					international
E.Bah 2011	database	Cancer survival in the Gambia, 1993-1997.	IARC scientific publications	Gambia	prospective follow-up	<500			small

Study ID	Identified through	Title	Journal	Country	Study Design	# of participants	# of data collection points	follow -up time	Reason for exclusion
El Imam 2006	database	Obstructive uropathy in Sudanese patients.	Saudi journal of kidney diseases and transplantation	Sudan	cross-sectional				cross-sectional
Elsharif 2011	database	Mortality rate of patients with end stage renal disease on regular hemodialysis: a single center study.	Saudi journal of kidney diseases and transplantation	Sudan	cross-sectional				cross-sectional
Emma-Okon 2014	database	Lipid profile in an apparently healthy Nigerian population.	The Nigerian postgraduate medical journal	Nigeria	cross-sectional				cross-sectional
Ferrari 2015	database	Geographical variations in the prevalence and management of cardiovascular risk factors in outpatients with CAD: Data from the contemporary CLARIFY registry.	European journal of preventive cardiology	Western/ Central Europe, Canada/South Africa/Australia/UK, Eastern Europe, Central/South America, Middle East, East Asia, India					international

Study ID	Identified through	Title	Journal	Country	Study Design	# of participants	# of data collection points	follow-up time	Reason for exclusion
Firnhaber 2016	database	Prospective One Year Follow Up of HIV Infected Women Screened for Cervical Cancer Using Visual Inspection with Acetic Acid, Cytology and Human Papillomavirus Testing in Johannesburg South Africa.	PloS one	South Africa	prospective follow-up	>500	1		1 data collection point
Gatimu 2016	database	Prevalence and determinants of diabetes among older adults in Ghana	BMC public health	Ghana	cross-sectional analysis of cohort data	<500			small
Geubbels 2015	database	Health & Demographic Surveillance System Profile: The Ifakara Rural and Urban Health and Demographic Surveillance System (Ifakara HDSS)	Int J Epidemiol	Tanzania	cross-sectional				cross-sectional
Gildner 2014	database	Sleep duration, sleep quality, and obesity risk among older	American journal of human biology	China, Ghana, India, Mexico,	international study				international

Study ID	Identified through	Title	Journal	Country	Study Design	# of participants	# of data collection points	follow-up time	Reason for exclusion
		adults from six middle-income countries: findings from the study on global AGEing and adult health (SAGE).		Russia, South Africa					
Gildner 2014	database	Sleep duration, sleep quality, and obesity risk among older adults from six middle-income countries: findings from the study on global AGEing and adult health (SAGE).	American journal of human biology	China, Ghana, India, Mexico, Russia, South Africa					international
Githinji 2017	database	Lung Function in South African Adolescents Infected Perinatally with HIV and Treated Long-Term with Antiretroviral Therapy	Ann Am Thorac Soc	South Africa	cross-sectional				cross-sectional
Hambridge 2017	database	Anthropometric indices for non-pregnant women of childbearing age differ widely among four low-	BMC public health	Equateur, Guatemala, India, Pakistan, Democratic	cross-sectional				cross-sectional

Study ID	Identified through	Title	Journal	Country	Study Design	# of participants	# of data collection points	follow-up time	Reason for exclusion
		middle income populations.		Republic of the Congo					
Hambridge 2017	database	Anthropometric indices for non-pregnant women of childbearing age differ widely among four low-middle income populations.	BMC public health	Equateur, Guatemala, India, Pakistan, Democratic Republic of the Congo					international
Hennild 2016	database	Prevalence of impaired glucose tolerance and other types of dysglycaemia among young twins and singletons in Guinea-Bissau.	BMC endocrine disorders	Guinea-Bissau	cross-sectional				cross-sectional
Jean-Luc Gradidge 2016	database	Metabolic and Body Composition Risk Factors Associated with Metabolic Syndrome in a Cohort of Women with a High Prevalence of	PloS one	South Africa	cross-sectional analysis of cohort data	<500			small

Study ID	Identified through	Title	Journal	Country	Study Design	# of participants	# of data collection points	follow-up time	Reason for exclusion
		Cardiometabolic Disease							
Jinabhai 2005	database	Changing patterns of under- and over-nutrition in South African children-future risks of non-communicable diseases.	Annals of tropical paediatrics	South Africa	cross-sectional				cross-sectional
K.Ackoundou-N'Guessan 2014	database	Uncontrolled hypertension in chronic kidney disease in patients from black Africa admitted in a renal unit: a retrospective analysis.	Nephrology therapy	Ivory Coast	retrospective analysis	<500			small
Kahn 2012	database	Profile: Agincourt health and socio-demographic surveillance system	Int J Epidemiol	South Africa	cross-sectional				cross-sectional
Kayima 2015	database	Determinants of hypertension in a young adult Ugandan population in	BMC public health	Uganda	cross-sectional				cross-sectional

Study ID	Identified through	Title	Journal	Country	Study Design	# of participants	# of data collection points	follow -up time	Reason for exclusion
		epidemiological transition-the MEPI-CVD survey.							
Kimbally-Kaky 2008	database	[Rheumatic heart disease in schoolchildren in Brazzaville].		Congo	cross-sectional				cross-sectional
Kisoli 2015	database	Levels of functional disability in elderly people in Tanzania with dementia, stroke and Parkinson's disease.	Acta neuropsychiatrica	Tanzania	cross-sectional				cross-sectional
Lane 2003	database	Apolipoprotein E and mortality in African-Americans and Yoruba.	Journal of Alzheimer's disease	USA, Nigeria					international
Makgae 2007	database	Somatotype and blood pressure of rural South African children aged 6-13 years: Ellisras longitudinal growth and health study.	Annals of human biology	South Africa	cross-sectional analysis of cohort data	>500	1		1 data collection point
Malete 2013	database	Body image and weight control in South Africans 15	BMC Public Health	South Africa	cross-sectional				cross-sectional

Study ID	Identified through	Title	Journal	Country	Study Design	# of participants	# of data collection points	follow -up time	Reason for exclusion
		years or older: SANHANES-1							
Mancuso 2016	database	The contribution of rare variation to prostate cancer heritability.	Nature genetics	Uganda	cross-sectional				cross-sectional
Ocheni 2008		Changing pattern of childhood malignancies in Eastern Nigeria.	West African journal of medicine	Nigeria	retrospective anlaysis of longitudinal surveys	<500			small
Onyango 2011	database	Post-partum weight change patterns in the WHO Multicentre Growth Reference Study.	Maternal & child nutrition	Brazil, Ghana, India, Norway, Oman, US		<500			small
Padalko 2015	database	Evaluation of the clinical significance of human papillomavirus (HPV) 53.	European journal of obstetrics, gynecology, and reproductive biology	Belgium, DRCongo					international
Patel 2005	database	Ischemic stroke in young HIV-positive patients in Kwazulu-Natal, South Africa.	Neurology	South Africa	cohort study	<500			small
Pedro 2016	database	CardioBengo study protocol: a population based cardiovascular longitudinal study	BMC public health	Angola	protocol for cohort	<500			small

Study ID	Identified through	Title	Journal	Country	Study Design	# of participants	# of data collection points	follow-up time	Reason for exclusion
		in Bengo Province, Angola.							
Phillips-Howard 2014	database	Deaths ascribed to non-communicable diseases among rural Kenyan adults are proportionately increasing: evidence from a health and demographic surveillance system, 2003-2010.	PloS one	Kenya	cross-sectional				cross-sectional
Phillips-Howard 2014	database	Deaths ascribed to non-communicable diseases among rural Kenyan adults are proportionately increasing: evidence from a health and demographic surveillance system, 2003-2010.	PloS one	Kenya	cross-sectional				cross-sectional

Study ID	Identified through	Title	Journal	Country	Study Design	# of participants	# of data collection points	follow-up time	Reason for exclusion
Pieters 2010	database	Triglyceride concentration and waist circumference influence alcohol-related plasminogen activator inhibitor-1 activity increase in black South Africans.	Blood coagulation & fibrinolysis	South Africa	cross-sectional analysis of cohort data	<500			small
R.Cooper 2015	database	Elevated hypertension risk for African-origin populations in biracial societies: modeling the Epidemiologic Transition Study.	Journal of hypertension	USA, Jamaica, Ghana, South Africa, Seychelles	cross-sectional analysis of cohort data	<500			small
Raji 2017	database	Awareness of hypertension and its impact on blood pressure control among elderly nigerians: report from the Ibadan study of aging.	The Pan African medical journal	Nigeria	community-based cohort	<500			small

Study ID	Identified through	Title	Journal	Country	Study Design	# of participants	# of data collection points	follow -up time	Reason for exclusion
Ramoshaba 2016	database	Components of Height and Blood Pressure among Ellisras Rural Children: Ellisras Longitudinal Study.	International journal of environmental research and public health	South Africa	cross-sectional analysis of longitudinal data	>500	1		1 data collection point
Rice 2006	database	Meta-analysis of genome-wide scans for blood pressure in African American and Nigerian samples. The National Heart, Lung, and Blood Institute GeneLink Project.	American journal of hypertension	Nigeria, USA	cross-sectional				cross-sectional
S.Abebe 2016	database	Diabetes mellitus among HIV-infected individuals in follow-up care at University of Gondar Hospital, Northwest Ethiopia.	BMJ open	Ethiopia	cross-sectional				cross-sectional
S.Abera 2017	database	Social determinants of adult mortality from non-communicable diseases in	PloS one	Ethiopia	cross-sectional				cross-sectional

Study ID	Identified through	Title	Journal	Country	Study Design	# of participants	# of data collection points	follow-up time	Reason for exclusion
		northern Ethiopia, 2009-2015: Evidence from health and demographic surveillance site.							
Safaeian 2007	database	Comparability of self-collected vaginal swabs and physician-collected cervical swabs for detection of human papillomavirus infections in Rakai, Uganda.	Sexually transmitted infections	Uganda	cross-sectional				cross-sectional
Schwartz 2009	database	Global meta-analysis of the C-11377G alteration in the ADIPOQ gene indicates the presence of population-specific effects: challenge for global health initiatives.	The pharmacogenomics journal	South Africa, Cuba, Germany	meta-analysis of cohorts	<500			small
Sliwa 2008	database	Spectrum of heart disease and risk factors in a black urban	Lancet	South Africa	cross-sectional				cross-sectional

Study ID	Identified through	Title	Journal	Country	Study Design	# of participants	# of data collection points	follow-up time	Reason for exclusion
		population in South Africa (the Heart of Soweto Study): a cohort study.							
Sliwa 2012	database	Different lipid profiles according to ethnicity in the Heart of Soweto study cohort of de novo presentations of heart disease.	Cardiovascular journal of Africa	South Africa	cross-sectional				cross-sectional
Sliwa 2012	database	Contribution of the human immunodeficiency virus/acquired immunodeficiency syndrome epidemic to de novo presentations of heart disease in the Heart of Soweto Study cohort.	European heart journal cardiovascular Imaging	South Africa	prospective, clinical registry	>500	1		1 data collection point
Stewart 2008	database	Predominance of heart failure in the Heart of Soweto Study cohort: emerging challenges for	Circulation	South Africa	prospective, clinical registry	>500	1		1 data collection point

Study ID	Identified through	Title	Journal	Country	Study Design	# of participants	# of data collection points	follow-up time	Reason for exclusion
		urban African communities.							
Stewart 2011	database	Standing at the crossroads between new and historically prevalent heart disease: effects of migration and socio-economic factors in the Heart of Soweto cohort study.	European Heart journal	South Africa	prospective, clinical registry	>500	1		1 data collection point
Stewart 2011b	database	The clinical consequences and challenges of hypertension in urban-dwelling black Africans: insights from the Heart of Soweto Study.		South Africa	prospective, clinical registry	>500	1		1 data collection point
Stewart 2011c	database	A not-so-rare form of heart failure in urban black Africans: pathways to right heart failure in the Heart of Soweto Study cohort.		South Africa	prospective, clinical registry	>500	1		1 data collection point

Study ID	Identified through	Title	Journal	Country	Study Design	# of participants	# of data collection points	follow-up time	Reason for exclusion
Urena-Torres 2011	database	Association of kidney function, vitamin D deficiency, and circulating markers of mineral and bone disorders in CKD.	American journal of kidney diseases	Sudan	cross-sectional				cross-sectional
van der Sande 2001	database	Geographical variation in prevalence of hypertension within The Gambia.	Journal of human hypertension	The Gambia	follow-up study	<500			
Victora 2008	database	Maternal and child undernutrition: consequences for adult health and human capital.	Lancet	Brazil, Guatemala, India, Phillipines, South Africa	analysis of prospective cohorts	<500			

STable 4a: Characteristics of included studies with only children and young adults aged <20 years, sampled from general populations

**Name of cohort and reference**	**Mandela's children: The 1990 Birth to Twenty Cohort (BT20)**[18]–[35]	**Seychelles Child Development Study**[36]–[40]	**The Lungwena Child Survival Study** [32],[41]–[45]
**Country**	South Africa	Seychelles	Malawi
**Study design**	birth cohort	cohort study	birth cohort
**Start date**	1990	1989	1995
**Total duration of study**	28yrs	20yrs	23yrs
**# of data collection points**	15	≥4	3
**Main focus**	child development	selected by MOH to focus on four important stages in development	child survival
**Funding of study**	Wellcome Trust, University of Witwatersrand, South African MRC, South African Human Science Research Council	McCabe Fund of the University of Pennsylvania, Rotary Club of Victoria, NIH, MOH of Seychelles, National Institutes of Environmental health Sciences, National Centre for Research Resources	Academy of Finland, the Emil Aaltonen Foundation, the Foundation for Paediatric Research in Finland, the Medical Research Fund of Tampere University Hospital, the Research Foundation of Mannerheim League for Child Welfare, the Research Foundation of the University of Tampere
**Stored samples**	**DNA** (n=2200 child samples; 2200 one biological parent)	none reported	none reported
**Potential for data access?**	No	not reported	not reported

MRC: Medical Research Council; NIH: National Institutes of Health; MOH: Ministry of Health

STable 4b: Characteristics of included studies with mixed (children and adults) populations, sampled from general and from clinical populations

Name of cohort and reference	**Rakai Community Cohort Study (RCCS)**[47]–[50]	**Kiang West Longitudinal Population Study**[51]–[53]	**The General Population Cohort (GPC)**[49],[54]–[57]	**Bukavu Observ Study** [58]-[59]	**Ellisras Longitudinal Study** [62]-[66]	**Mildmay Uganda Cohort**[67],[68]	**Prospective cohort study in Sudan**[69]
**Country**	Uganda	Gambia	Uganda	DR Congo	South Africa	Uganda	Sudan
**Study design**	open, population-based cohort	longitudinal study	community-based open cohort	observational cohort	longitudinal study	clinical cohort	longitudinal surveys
**Start date**	1994	1980 (biobank 2012)	1989	2012	1996	2004	2009
**Total duration of study**	11yrs	38yrs	22yrs	3yrs	7rs	7yrs	15mo
**# of data collection points**	11	≥12	22	3	13	~42	2
**Main focus**	HIV incidence	life-course longitudinal nutritional and health phenotypes; integrated within Kiang West DSS, KEMRes, and Keneba Biobank	HIV, NCDs, genetics	prevalence of NCDs	growth and health	to monitor clinical care and outcomes of HIV among patients on treatment	to evaluate the survival of haemodialysis patients
**Funding of study**	National Institute of Mental Health, National Institute of Allergy and Infectious Diseases, National Institute of Child Health and Development, National Institute for Allergy and Infectious Diseases Division of Intramural Research, NIH, Bill	MRC UK, UK Department for International Development	MRC UK, Wellcome Trust	Flemish Inter-University Council	VU University Medical Centre, University of Limpopo South Africa, National Research Foundation, MRC of South Africa, University of the North South Africa	Canadian Institutes for Health Research, Grand Challenges Canada, President's Emergency Plan for AIDS Relief	not reported

Name of cohort and reference	**Rakai Community Cohort Study (RCCS)**[47]–[50]	**Kiang West Longitudinal Population Study**[51]–[53]	**The General Population Cohort (GPC)**[49],[54]–[57]	**Bukavu Observ Study** [58]-[59]	**Ellisras Longitudinal Study** [62]-[66]	**Mildmay Uganda Cohort**[67],[68]	**Prospective cohort study in Sudan**[69]
	& Melinda Gates Foundation, John Hopkins University Centre for AIDS Research						
**Stored samples**	**venous blood samples** and **archived serum samples** stored at -70C	**DNA** extracted in 2002-2003 using standard salting-out method according to DNA Bank	**DNA** extracted from serum samples (available for 8000) venous blood samples stored at -80C	none reported	none reported	none reported	none reported
**Potential for data access?**	yes	yes	yes	yes	not reported	yes	not reported

DSS: Demographic Surveillance Site; KEMRes: Keneba Electronical Medical Records System; MRC: Medical Research Council; NIH: National Institutes of Health

STable 4c: Characteristics of included studies with only adults aged >18 yrs, sampled from general populations

Name of cohort and reference	**African Collaborative Centre for Microbiome and Genomics Research's (ACCME's) HPV and Cervical Cancer Study**[70]	**Africa Wits-INDEPTH partnership for Genomic Studies (AWI-Gen)**[71],[72]	**Health and Aging in Africa: a Longitudinal Study of an INDEPTH Community in South Africa (HAALSI)**[73]–[75]	**Non-Communicable Disease Intervention Programme**[76]–[83]	**Dar es Salaam Urban Cohort Hypertension Study (DUCS-HTN)**[84]	**Ibadan Study of Aging**[85]–[88]	**Prospective Cohort in Democratic Republic of Congo**[89]	**The Benin Study**[90],[91]
**Country**	Nigeria	Burkina Faso, Ghana, Kenya, South Africa	South Africa	Mauritius	Tanzania	Nigeria	DR Congo	Benin
**Study design**	cohort study	longitudinal study	population-based longitudinal cohort	longitudinal population-based surveys	cohort study	community-based cohort	prospective cohort	longitudinal observational study
**Start date**	2014	2013	2014	1987	2014	2003	2004	2005
**Total duration of study**	24mo	5yrs	4yrs	11yrs	1yr	4yrs	4yrs	5yrs
**# of data collection points**	5	1 (next planned in 2019)	≥8	3	2	2	8	3
**Main focus**	environmental, microbiomic, genetic and epigenetic factors associated with persistent HPV infection	genetic and environmental factors that contribute to cardiometabolic disease in African populations	CVDs, HIV, cognitive function and dementia	NCD intervention	prevalence, risk factors, and barriers to diagnosis and treatment for hypertension in Dar es Salaam	functioning and disability of elderly persons	type 2 diabetes incidence	evolution of cardiometabolic risk
**Funding of study**	NIH	NIH	National Institute on Aging	US NIH	Centre for Global Demography of Ageing	Wellcome Trust	LOMO Medical Clinic, Regional Office of WHO for Africa	Canadian International Development Agency

**Name of cohort and reference**	**African Collaborative Centre for Microbiome and Genomics Research's (ACCME's) HPV and Cervical Cancer Study**[70]	**Africa Wits-INDEPTH partnership for Genomic Studies (AWI-Gen)**[71],[72]	**Health and Aging in Africa: a Longitudinal Study of an INDEPTH Community in South Africa (HAALSI)**[73]–[75]	**Non-Communicable Disease Intervention Programme**[76]–[83]	**Dar es Salaam Urban Cohort Hypertension Study (DUCS-HTN)**[84]	**Ibadan Study of Aging**[85]–[88]	**Prospective Cohort in Democratic Republic of Congo**[89]	**The Benin Study**[90],[91]
**Stored samples**	**cervical biopsy** samples stored as fresh frozen and paraffin embedded; **venous blood samples** separated into plasma, serum, buffy coat, red blood cells, and clot and stored at -80C	**serum, plasma, urine aliquots** frozen at -80C	none reported	none reported	none reported	none reported	none reported	none reported
**Potential for data access?**	`	yes	yes	not reported	not reported	yes	not reported	not reported

NIH: National Institute of Health; CVD: Cardiovascular Diseases

STable 4d: Characteristics of included studies with only adults aged >18yrs, sampled from occupational and clinical populations

Name of cohort and reference	**Basotho Gold Miners Cohort**[92],[93]	**Themba Lethu Clinical (TLC) Cohort Study**[94],[95]	**Cohort of HIV-infected, ART-naïve adults**[96],[97]	**Operational cohort of treatment naïve HIV-infected men and women**[98],[99]	**Prospective cohort of nulliparous women**[100],[101]	**The African Breast Cancer Disparities in Outcomes (ABC-DO) Study**[102],[103]	**HPV in Africa Research Partnership (HARP) Study**[104]–[106]	**The Complications of Long-Term Antiretroviral Therapy among HIV-positive Ugandan adults (CoLTART) study**[107]–[109]
**Country**	Lesotho	South Africa	Zambia	South Africa	Nigeria	Namibia, Nigeria, South Africa, Uganda, Zambia	Burkina Faso, South Africa	Uganda
**Study design**	occupational cohort study	clinical cohort study	clinical cohort study	clinical cohort study	prospective clinical cohort	prospective hospital-based study	prospective clinical cohort	prospective clinical cohort
**Start date**	1999	2004	2004	2003	2006	2014	2011	2013
**Total duration of study**	1yr	14yrs	3yrs	8yrs	3yrs	3yrs	18mo	18mo
**# of data collection points**	2	≥14	2	≥15	2	9	≥2	3
**Main focus**	health of gold miners	HIV	effects of ART	a wellness clinic, which has provided pre-antiretroviral therapy care and support for HIV-infected adults in Soweto	health outcomes of pregnant women	overall survival, impact on quality of life, and delays along the journey from symptom discovery to diagnosis and treatment of breast cancer in SSA	evaluation of cervical cancer screening approaches among women living with HIV	platform for studying the complications of long-term ART among Ugandan HIV-positive adults
**Funding of study**	AngloGold Corporation	USAID, National Institute of Allergy and Infectious Diseases, National	Elizabeth Glaser Paediatric AIDS Foundation from the US Centres for Disease Control and	USAID South Africa	Gates Institute of the Johns Hopkins University, MacArthur	Susan G Komen for the Cure Foundation, IARC	European Commission 7th Framework Programme	UK MRC, UK Department for International Development

Name of cohort and reference	**Basotho Gold Miners Cohort**[92],[93]	**Themba Lethu Clinical (TLC) Cohort Study**[94],[95]	**Cohort of HIV-infected, ART-naïve adults**[96],[97]	**Operational cohort of treatment naïve HIV-infected men and women**[98],[99]	**Prospective cohort of nulliparous women**[100],[101]	**The African Breast Cancer Disparities in Outcomes (ABC-DO) Study**[102],[103]	**HPV in Africa Research Partnership (HARP) Study**[104]–[106]	**The Complications of Long-Term Antiretroviral Therapy among HIV-positive Ugandan adults (CoLTART) study**[107]–[109]
		Institute of Child Health and Development, South African Mission of the US Agency for International Development	Prevention, Doris Duke Charitable Foundations, NIH, University of Alabama at Birmingham Centre for AIDS Research		Multidisciplinary Resarch grant			
**Stored samples**	none reported	none reported	none reported	none reported	none reported	formalin-fixed paraffin embedded tumour blocks	none reported	**whole blood** for genomics studies stored at -80C
**Potential for data access?**	yes	yes	not reported	yes	not reported	yes	not reported	yes

NIH: National Institute of Health; MRC: Medical Research Council: IARC: International Agency for Research on Cancer; ART: antiretroviral therapy; WIHS: Women's Interagency HIV Study

STable 4d continued: Characteristics of included studies with only adults aged >18yrs, sampled from occupational and clinical populations

Name of cohort and reference	**Prospective cohort study in Ghana**[110]	**Rwanda Women's Interassociation Study and Assessment (RWISA)**[111]–[114]	**Prospective cohort study in Benin**[115]	**Uganda AIDS Rural Treatment Outcomes (UARTO) cohort**[116]–[118]
**Country**	Ghana	Rwanda	Benin	Uganda
**Study design**	prospective clinical cohort	observational prospective clinical cohort	prospective clinical cohort	population-based prospective cohort
**Start date**	2012	2005	not reported	2005
**Total duration of study**	2yrs	13yrs	1yr	10yrs
**# of data collection points**	2	26	5	≥2
**Main focus**	perinatal outcomes of women with pregnancies complicated by pregnancy-induced hypertension, chronic hypertension and pre-eclampsia	effectiveness and toxicity of ART	contrasting post-partum health of women	effects of ART
**Funding of study**	Ridge Regional Hospital Out Patient Clinic, Maamobi General Hospital	National Institute of Allergy and Infectious Diseases, NIH, National Institute of Diabetes and Digestive and Kidney Disease, Chicago WIHS	WHO	NIH, Doris Duke Charitable Foundation, the Sullivan Family Foundation, the Canada-Africa Prevention Trials network
**Stored samples**	none reported	none reported	none reported	none reported
**Potential for data access?**	not reported	no	not reported	not reported

NIH: National Institute of Health

STable 5a: Participant characteristics of included studies with children and young adults aged <20 years, sampled from general populations

Name of cohort	Birth to Twenty	Seychelles Child Development Study	The Lungwena Child Survival Study
Sampling frame	general	public and private schools	general
Inclusion criteria	included all singletons born to women resident in Soweto-Johannesburg during 7wk enrolment period (≥6wks spent in area)	included all children in four grades attending all public and private schools of Seychelles	included live-born singleton offspring of a cohort of women
Subject flow (# eligible # enrolled)	eligible 5449<br>enrolled 3273	eligible 933<br>enrolled 779	eligible 795<br>enrolled 729
Sample size at final evaluation	6291	549	314
Age (mean)	birth-18yrs	6mo - 20yrs	birth-17yrs
Possible to stratify on age?	yes	yes	yes
Sex (%)	males (48)<br>females (52)	males (48)<br>females (52)	males (58)<br>females (42)
Race/ethnicity	African (78)<br>White (6)<br>Coloured (12)<br>Indian (4)	not reported	mostly Islamic
Rural/urban location (%)	urban (100)	not reported	rural (100)
Comparison groups	hypertensive (124) vs non-hypertensive (714)	none reported	stunted at 12mo (71%) vs not stunted at 12mo (29%)

STable 5b: Participant characteristics of included studies with mixed (children and adults) populations, sampled from general and clinical populations

Name of cohort	RCCS	Kiang West Longitudinal Population Study	The General Population Cohort	Bukavu Observ Study	Ellisras Longitudinal Study	Mildmay Uganda Cohort	Prospective cohort study in Sudan
**Sampling frame**	general population	residents across 36 villages within rural Kiang West HDSS	general population	general population	22 schools (10 pre-schools, 12 primary)	all patients who initiated combination ART at Mug	patients at haemodialysis centres in Khartoum
**Inclusion criteria**	included all individuals aged 15-49yrs residing for ≥6mo in agrarian and trading communities, 1mo intention to stay longer	not reported	included individuals residing ≥3mo within study area and aged ≥13yrs	all adults asked to participate	not reported	included patients beginning therapy at 13yrs with ≥ 2 BP measurements available	included all patients on haemodialysis in these centres
**Subject flow (# eligible # enrolled)**	eligible 53933 enrolled 33438	eligible not reported enrolled 14000 (biobank 9000)	eligible 11018 enrolled 7725	eligible 4345 enrolled 4237	eligible not reported enrolled 2225	eligible 8835 enrolled 5563	eligible 2072 enrolled 1011
**Sample size at final evaluation**	17119	14846	7830	3962	771	5563	967
**Age (mean)**	15-49yrs (23)	5-50yrs	≥13yrs (44)	≥15yrs (34)	3-28yrs	≥13yrs (35)	6-95 (45)
**Possible to stratify on age?**	yes	yes	yes	yes	yes	yes	no
**Sex (%)**	males (46) females (54)	males (43) females (57)	males (44) females (56)	males (44) females (56)	males (52) females (48)	males (33) females (67)	males (567) females (33)
**Race/ethnicity**	not reported	Mandinka (80) Fula (16) Jola (2) Other (3)	Muganda (75) Rwandese/Burundi (19) Other (6)	not reported	not reported	not reported	not reported
**Rural/urban location (%)**	rural (84) urban (16)	rural (100)	rural (100)	rural (100)	rural (100)	not reported	not reported

**Name of cohort**	**RCCS**	**Kiang West Longitudinal Population Study**	**The General Population Cohort**	**Bukavu Observ Study**	**Ellisras Longitudinal Study**	**Mildmay Uganda Cohort**	**Prospective cohort study in Sudan**
**Comparison groups**	HIV+ (3488) vs HIVø (13361)	none reported	low HDL (5285) vs normal HDL (2101)	malnourished (269) vs normal weight (3003) vs overweight (551) vs obese (139)	none reported	none reported	adults (968) vs paediatrics (43)

HDSS: Health & Demographic Surveillance System; HIV ø: HIV negative; ART: antiretroviral therapy; MUg: Mildmay Uganda Clinic; BP: blood pressure

STable 5c: Participant characteristics of included studies with only adults aged >18 yrs, sampled from general populations

Name of cohort	ACCME's HPV and Cervical Cancer Study	AWI-Gen	HAALSI	NCD Intervention Programme	DUCS-HTN	Ibadan Study of Aging	Prospective Cohort in DRC	The Benin Study
**Sampling frame**	42 districts in Abuja	six INDEPTH member HDSS centres	adults in Agincourt HDSS	10 geographically defined areas	seven neighbourhoods within HDSS	general population	drawn from cross-sectional study	Cotonou, Ouidah
**Inclusion criteria**	included females ≥18yrs, history of sexual intercourse, no previous history of cervical abnormalities, cervical cancer or total abdominal hysterectomy; excluded HIV+ women	included unrelated participants 40-60yrs resident in areas served by the HDSS centres; excluded pregnant women, recent immigrants (<10yrs)	included ≥40yrs, permanently living in the study site for 12mo preceding 2013 HDSS census	included all adults 25-74yrs living within defined areas	included HDSS participants ≥40yrs, living in 1 of 2 neighbourhoods	included ≥65yrs, fluent in Yoruba	excluded participants ≤40yrs, cases of known diabetes, insufficient baseline data	excluded prior diagnosis of hypertension, diabetes, CVDs
**Subject flow (# eligible # enrolled)**	eligible not reported enrolled 11400	eligible not reported enrolled 10696	eligible 12872 enrolled 6281	eligible 5910 enrolled 5083	eligible 4896 enrolled 2290	eligible 2900 enrolled 2152	eligible 960 enrolled 807	eligible not reported enrolled 541
**Sample size at final evaluation**	5349	not reported	5059	6291	1752	1330	not reported	416
**Age (mean)**	≥18yrs (39)	40-60yrs (50)	≥40yrs (62)	25-74yrs (43)	≥40yrs (51)	≥65yrs (77)	>40yrs	24-60yrs
**Possible to stratify on age?**	yes	yes	yes	no	yes	yes	no	yes
**Sex (%)**	females (100)	males (45) females (55)	males (46) females (54)	males (46) females (54)	males (44) females (56)	males (46) females (54)	males (54) females (46)	males (50) females (50)
**Race/ethnicity**	Hausa tribe (7.5) Ibo tribe (22.0) Yoruba tribe (11.5)	10 SA ethnicities (47) 8 BF ethnicities (19) 8 Ghana	SA (70) Mozambican/other (30)	Asian Indian (68) Chinese (3) Creoles (27)	not reported	not reported	not reported	not reported

Name of cohort	ACCME's HPV and Cervical Cancer Study	AWI-Gen	HAALSI	NCD Intervention Programme	DUCS-HTN	Ibadan Study of Aging	Prospective Cohort in DRC	The Benin Study
	Other tribes (59.0)	ethnicities (18) 13 Kenya ethnicities (18)						
**Rural/urban location (%)**	rural (12) semi-rural (46) urban (42)	not reported	rural (100)	not reported	urban (100)	rural (34) urban (26) semi-urban (40)	not reported	rural (31) urban (37) semi-urban (32)
**Comparison groups**	HPVø (6727) vs HPV+ (4773)	SA (4848) BF (2079) Ghana (1988) Kenya (1942)	HIVø (3279) vs HIV+ (1134) dyslipidaemic (2860) vs non-dyslipidaemic (1387)	Mauritian Indian (9024) vs Mauritian Creole (1075) diabetic (628) vs non-diabetic (3317)	none reported	major depressive disorder cases (134) vs non (2015) hypertensive (973) vs normotensive (496)	with metabolic syndrome (393) vs without metabolic syndrome (414)	insulin resistant (106) vs non-insulin resistant (310)

ART: antiretroviral therapy; MUg: Mildmay Uganda Clinic; BP: blood pressure; CC: cervical cancer; HPVø: HPV negative; HIVø: HIV negative; HDSS: Health & Demographic Surveillance Systems; CVD: cardiovascular diseases

STable 5d: Participant characteristics of included studies with only adults aged >18 yrs, sampled from occupational and clinical populations

**Name of cohort**	**Basotho Gold Miners Cohort**	**TLC Cohort Study**	**Cohort of HIV-infected, ART-naïve adults**	**Operational cohort of treatment naïve HIV-infected men and women**	**Prospective cohort of nulliparous women**	**ABC-DO Study**	**HARP Study**	**CoLTART Study**
**Sampling frame**	a single mine	1 clinic	primary care facilities within Lusaka	Perinatal HIV Research Unit, urban Wellness clinic	three hospitals	secondary and tertiary hospitals	HIV outpatient clinic in BF and HIV treatment centres in SA	DART Trial, RCC, GPC, Entebbe Pre-ART cohort
**Inclusion criteria**	included miners originally from Lesotho laid off from a single mine	included HIV+ patients seen at TLC, ≥18yrs, either treatment-naïve or switched onto ART regimen containing 300mg tenofovir daily since Apr 2004 and by Oct 2011	included adults ≥15yrs, treatment-naïve/newly initiating ART, with baseline creatinine	included adults ≥18yrs	included nulliparous women at gestational ages <20wks; excluded pre-existing conditions (including hypertension), previous spontaneous abortions	included women ≥18yrs presenting with new primary BC diagnosis; excluded women already receiving treatment, benign lesions	included HIV-1 seropositivity, aged 25-50yrs, resident in the city; excluded history of prior CC treatment, previous hysterectomy, pregnant or <8wks post-partum, women with CIN2+ at baseline from follow-ups	included HIV+ adults ≥18yrs, on ART/eligible to initiate, not participating in another study; excluded those too sick to undergo study procedures
**Subject flow (# eligible # enrolled)**	eligible 779<br>enrolled 624	eligible not reported<br>enrolled 30000	eligible 36289<br>enrolled 25779	eligible not reported<br>enrolled 3635	eligible not reported<br>enrolled 3000	eligible 2100<br>enrolled 2100	eligible 1473<br>enrolled 1077	eligible 1108<br>enrolled 1095
**Sample size at final evaluation**	553	ongoing accrual	9633	3308	2630	2100	963	not reported
**Age (mean)**	30-59yrs (49)	>18yrs	>15yrs	≥18 (33)	(27)	≥18yrs	25-50yrs<br>BF (36:median)<br>SA (34:median)	≥18 (45)
**Possible to stratify on age?**	no	yes	no	no	yes	no	no	yes
**Sex (%)**	males (100)	males (36)<br>females (64)	males (40)<br>females (60)	males (22)<br>females (78)	females (100)	females (100)	females (100)	males (35)<br>females (65)

Name of cohort	Basotho Gold Miners Cohort	TLC Cohort Study	Cohort of HIV-infected, ART-naïve adults	Operational cohort of treatment naïve HIV-infected men and women	Prospective cohort of nulliparous women	ABC-DO Study	HARP Study	CoLTART Study
**Race/ethnicity**	not reported	SA (91)<br>non-SA (9)<br>other (0)	not reported	not reported	not reported	Namibia - black (22) Namibia - non-black (6)<br>Nigeria (21)<br>SA - black (19)<br>SA - non-black (2)<br>Uganda (22)<br>Zambia (88)	not reported	not reported
**Rural/urban location (%)**	rural (100)	urban (100)	urban (100)	not reported	not reported	rural (47)<br>urban (53)	not reported	rural (47)<br>urban (53)
**Comparison groups**	none reported	pre-ART (8217) vs ART (21101)	none reported	none reported	none reported	none reported	BF (583)<br>SA (494)	non-TDF ART (385) vs TDF ART (568)

ART: antiretroviral therapy; TDF: tenofovir' MUg: Mildmay Uganda Clinic; BP: blood pressure; BC: breast cancer; CC: cervical cancer; RCC: Rakai Community Cohort; GPC: General Population Cohort; DART: Development of Antiretroviral Therapy; HIVø: HIV negative;

STable 5d continued: Participant characteristics of included studies with only adults aged >18yrs, sampled from occupational and clinical populations

**Name of cohort**	**Prospective cohort study in Ghana**	**RWISA**	**Prospective cohort study in Benin**	**UARTO Cohort**
**Sampling frame**	two hospitals in Accra	grassroots women's orgs, HIV clinics	six referral facilities	Mbara Regional Referral Hospital
**Inclusion criteria**	excluded women with previously established chronic hypertension and <17wks pregnancy	included women ≥25yrs, present in Rwanda during 1994, no history of ART except single-dose nerivapine to prevent mother-to-child transmission	included women who gave birth over 12mo period	included treatment-naïve/newly initiating ART, ≥18yrs, living within 60km of clinic
**Subject flow (# eligible # enrolled)**	eligible not reported<br>enrolled 1010	eligible not reported<br>enrolled 936	eligible not reported<br>enrolled 709	eligible 1020<br>enrolled 500
**Sample size at final evaluation**	824	not reported	567	771
**Age (mean)**	≥18 (28)	≥25<br>HIV+ (35)<br>HIVø (42)	(26)	≥18yrs (34)
**Possible to stratify on age?**	no	yes	no	no
**Sex (%)**	females (100)	females (100)	females (100)	males (30) females (70)
**Race/ethnicity**	not reported	not reported	not reported	not reported
**Rural/urban location (%)**	rural (3)<br>Accra (77)<br>other urban (18)	not reported	semi-urban (100)	rural (100)
**Comparison groups**	normotensive (700) vs pregnancy induced hypertension (59) vs pre-eclampsia (14) vs chronic hypertension (2)	HIV+ (710) vs HIVø (226)	women with live birth (205) vs women with perinatal death (64) vs women with normal delivery (440)	3+ visits with high BP (73) vs no visits with high BP (157)

HIVø: HIV negative

STable 6a: Characteristics of chronic NCD data of included studies with children and young adults aged <20 years, sampled from general populations

Name of cohort	BT20	Seychelles Child Development Study	The Lungwena Child Survival Study
Hypertension definition	**hypertension** defined using age, sex and height standardised percentile tables for BP classification, where hypertension ≥95th percentile	**hypertension** not defined	**hypertension** not measured
Source of measurement	**BP** measured in triplicate using Omron M6 after participant had been sitting for 5min with resting intervals of 2min; average of three used to define status	**BP** measured in triplicate using Omron M5 after participant had been sitting for 50min; average of three used to define status	
# of time points measured	6	≥4	
Reporting of chronic NCD	point prevalence	none reported	
Estimate in total sample	14.8		
Associated risk factor(s) reported	SES, age, weight and height at 18yrs, relative weight gain		
Diabetes definition	**diabetes** defined as glucose ≥6.1mmol/L or receiving treatment for diabetes	**diabetes** not measured	**obesity** BMI cut-off charts for children used to calculate corresponding overweight cut-offs
Source of measurement	**glucose** concentrations measured using enzymatic methods on auto-analyser (Randox Daytona Clinical Analyser)		**weight** measured using spring scale until age 12mo, thereafter with digital bathroom scale to nearest 100g<br>**height** measured with locally constructed height boards to nearest 5mm in supine until able to stand
# of time points measured	2		≥2
Reporting of chronic NCD	point prevalence		point prevalence
Estimate in total sample	14.3		0
Associated risk factor(s) reported	gender, obesity		none reported
Obesity definition	**obesity** defined using WHO cut-off points for children 5-19yrs, where obesity BMIz ≥2.0SD from reference median	**obesity** defined using IOTF criteria corresponding to adult BMI of 30kg/m2 (British 1990 BMI SD >=+2.37 and >=+2.25 in boys and girls, respectively)	**obesity** not measured

Name of cohort	BT20	Seychelles Child Development Study	The Lungwena Child Survival Study
Source of measurement	**weight** measured using digital scale to nearest 1000g with participants wearing light clothing, no shoes<br>**height** measured using portable stadiometer to nearest 1mm with participants wearing light clothing, no shoes	**weight** measured using precision electronic scale (Seca 870)<br>**height** measured with fixed stadiometer (Seca 208)	
# of time points measured	15	≥4	
Reporting of chronic NCD	incidence	prevalence	
Estimate in total sample	20.5	3.8%	
Associated risk factor(s) reported	gender, overweight/ obesity in infancy/ toddlerhood and early childhood, early menarche	weight gain during first year of life, increased maternal BMI	
CKD definition	**CKD** not measured	**CKD** estimated GFR using Cockcroft-Gault formulae	**CKD** not measured
Source of measurement		fasting venous blood samples measured using standard enzymatic methods (Thermo Konelab 30 automatic analyser)	
# of time points measured		≥4	
Reporting of chronic NCD		mean (SD)	
Estimate in total sample		130.7(33.6)	
Associated risk factor(s) reported		none reported	

BMI: body mass index; BP: blood pressure; IOTF: International Obesity Task Force; SD: standard deviation; SES: socio-economic status; GFR: glomerular filtration rate; IOTF: International Obesity Task Force

STable 6b: Characteristics of chronic NCD data of included studies with mixed (children and adults) populations, sampled from general and clinical populations

Name of cohort	RCCS	Kiang West Longitudinal Population Study	GPC	Bukavu Observ Study	Ellisras Longitudinal Study	Mildmay Uganda Cohort	Prospective cohort study in Sudan
**Hypertension definition**	**high BP** not measured	**high BP in 5-18yrs** defined as SBP and/or DBP that is ≥95th percentile for sex, age, and age-specific height z scores in this population **high BP in >18yrs** defined as SBP ≥140mmHg and/or DBP ≥90mmHg and/or on antihypertensive medication the 3mo prior and/or being diagnosed as hypertensive in MRC Keneba clinical database	**hypertension** defined as SBP ≥140mmHg and/or DBP ≥90mmHg, or self-reported use of antihypertensive medications	**hypertension** defined as SBP ≥140mmHg and/or DBP ≥90mmHg, or self-reported use of antihypertensive medications	**hypertension** defined as average SBP and/or DBP that is ≥95th percentile for sex and age	**hypertension** defined as SBP ≥140mmHg and/or DBP ≥90mmHg on ≥2 visits	**target pre-haemodialysis BP** defined as SBP ≤140mmHg and/or DBP ≤90mmHg **target post-haemodialysis BP** defined as SBP ≤130mmHg and/or DBP ≤80mmHg
**Source of measurement**		**BP** measured in triplicate with Omron 705IT with resting intervals of 5min; average of second and third used to define status	**BP** measured in triplicate using Omron M4-1 after participant had been sitting for ≥15min with resting intervals of 5min; mean of second and third readings used to define status	**BP** measured in triplicate using Omron Hem 700`E after participant had been sitting for ≥5min; mean of three used to define status	**BP** measured in triplicate using electronic Micronta monitoring kit after participant had been sitting for ≥5min; average of three used to define status	**BP** measured using manual sphygmomanometer	**BP** measured prior, during, and after haemodialysis
**# of time points measured**		≥12	2	2	13	6	2
**Reporting of chronic NCD**		prevalence	age-adjusted prevalence	prevalence	prevalence	age-standardised prevalence	percentage achieving both pre- and post-haemodialysis BP

Name of cohort	RCCS	Kiang West Longitudinal Population Study	GPC	Bukavu Observ Study	Ellisras Longitudinal Study	Mildmay Uganda Cohort	Prospective cohort study in Sudan
**Estimate in total sample**		5-18yrs (8)<br>>18 (18)	7.1	2040%	1-11.4%	24.8%	20.3%
**Associated risk factor(s) reported**		age, BMI z-score, haemoglobin, high fasting glucose	lipids	none reported	weight-for-age z-scores, sum of four skinfolds, underweight	none reported	none reported
**Diabetes definition**	**diabetes** not measured	**diabetes** defined as fasting plasma glucose of ≥7mmol/L or receiving treatment for diabetes at MRC Keneba clinic	**diabetes** defined as HbA1c >6.5%	**diabetes** defined as fasting glucose ≥126mg/dol and/or taking diabetes medication	**pre-diabetes** defined as plasma glucose 5.6-6.9mmol/L	**diabetes** not measured	**diabetes** not measured
**Source of measurement**		**fasting plasma glucose** measured using Accu Check	**HbA1c** measured from whole-blood samples, analysis performed using Cobas Integra 400 plus chemistry analyser	**glucose concentration** analysed by glucose oxidase method using portable electronic monitor (Codefree)	**fasting plasma glucose** collected and stored for later analysis		
**# of time points measured**		≥12	2	1	≥1		
**Reporting of chronic NCD**		prevalence	age-adjusted prevalence	prevalence	prevalence		
**Estimate in total sample**		5-18yrs (0.8)<br>>18 (0.7)	1.1	280%	males (46)<br>females (50)		
**Associated risk factor(s) reported**		none reported	lipids	age, WC, BP	none reported		
**Obesity definition**	**obesity** not measured	**obesity in 5-18yrs** defined as weight-for-height z-scores >0.68<br>**obesity in >18yrs** defined as BMI ≥30	**obesity** defined as WC ≥94cm for men and ≥80cm for women	**obesity** defined as BMI ≥30<br>**abdominal obesity** defined as WC ≥94cm for both males and females	**overweight** defined according to the Cole et all BMI cut-off point	**obesity** not measured	**obesity** not measured

Name of cohort	RCCS	Kiang West Longitudinal Population Study	GPC	Bukavu Observ Study	Ellisras Longitudinal Study	Mildmay Uganda Cohort	Prospective cohort study in Sudan
**Source of measurement**		**weight** measured to nearest 10g using portable scales (Tanita WB100) **height** measured to nearest 0.1cm using stadiometer without footwear or headwear	**WC** measured in duplicate using Seca 201 Ergonomic Circumference Measuring Tape to noearest 0.1cm**;** mean of two used to define status	**weight** measured to nearest 100g using electronic scale (Tanita HD-325) with participants in light-weight clothing **height** measured with a SECA mesband 206cm **WC** measured using a tape measure to the nearest 0.5cm	**weight** measured to nearest 0.1kg using electric scale **height** measured to nearest 0.1cm using Martin anthropometer		
**# of time points measured**		≥12	2	3	13		
**Reporting of chronic NCD**		prevalence	age-adjusted prevalence	incidence	prevalence		
**Estimate in total sample**		5-18yrs (13) >18 (37)	10	535/100,000	males (-.3-4.9%) females (1.6-15.5%)		
**Associated risk factor(s) reported**		none reported	lipids	age, gender, PA	none reported		
**CKD definition**	**CKD** defined eGFR as mildly (60-89ml/min) or moderately reduced (<60ml/min)	**CKD** not measured	**CKD** not measured	**CKD** not measured	**CKD** not measured	**CKD** not measured	**CKD** not measured
**Source of measurement**	**eGFR** estimated using MDRD **creatinine concentration** measured from blood sera using rate-based enzymatic assay (Roche)						
**# of time points measured**	≥2						
**Reporting of chronic NCD**	rates of eGFR decline						

Name of cohort	RCCS	Kiang West Longitudinal Population Study	GPC	Bukavu Observ Study	Ellisras Longitudinal Study	Mildmay Uganda Cohort	Prospective cohort study in Sudan
**Estimate in total sample**	HIV positive (32)<br>HIV negative (20)						
**Associated risk factor(s) reported**	HIV infection						

eGFR: estimated glomerular filtration rate; BP: blood pressure; MRC: Medical Research Council; WC: waist-circumference; PA: physical activity: HIVø: HIV negative

STable 6c: Characteristics of chronic NCD data of included studies with adults aged >18 years, sampled from general populations

Name of cohort	ACCME's HPV and Cervical Cancer Study	AWI-Gen	HAALSI	NCD Intervention Programme	DUCS-HTN	Ibadan Study of Aging	Prospective Cohort in Democratic Republic of Congo	The Benin Study
**Hypertension definition**	**hypertension** defined as yes/no	**hypertension** defined as SBP ≥140 and/or DBP ≥90 and/or self-reported use of antihypertensive medication	**hypertension** defined as SBP ≥140 and/or DBP ≥90 and/or self-reported use of antihypertensive medication	**hypertension** defined as SBP ≥140 and/or DBP ≥90 and/or self-reported use of antihypertensive medication	**hypertension** defined as SBP ≥140 and/or DBP ≥90 and/or self-reported use of antihypertensive medication	**hypertension** defined as SBP ≥140 and/or DBP ≥90 and/or self-reported use of antihypertensive medication and/or previous diagnosis by physician	**hypertension** defined using IDF Europe criteria as SBP ≥130 and/or DBP ≥85	**hypertension** defined as SBP ≥130 and/or DBP ≥85
**Source of measurement**	**BP** measured in triplicate with OMRONVR BP760 after participate had been sitting for ≥15min with resting intervals of 1min	**BP** measured in triplicate with Omron M6 after participant had been sitting for ≥3min with resting intervals of 2min; average of second and third used to define status	**BP** measured in triplicate with Omron M6W after participant had been sitting for 5 \min with resting intervals of 2 \min; average of second and third used to define status	**BP** measured in duplicate using standard mercury sphygmomanometer after participant had been sitting for ≥5min; average of two used to define status	**BP** measured in triplicate with Omro M2/Beurer BMI 40 after participant had been sitting for ≥5min with resting intervals of 2min; average of two used to define status	**BP** measured in triplicate with Omro MS-2 after participant had been sitting for ≥5min with resting intervals of 5min; average of three used to define status	**BP** measured in triplicate with standard mercury sphygmomanometer after participant had been sitting for ≥30min with resting intervals of 2min; average of second and third used to define status	**BP** measured in duplicate with standard mercury sphygmomanometer after participant had been sitting for ≥10min with resting intervals of 2min; average of two used to define status
**# of time points measured**	5	1	≥8	3	1 (1/5 with 2)	1	1	3
**Reporting of chronic NCD**	baseline prevalence	mean prevalence	prevalence	incidence	prevalence	12mo prevalence	point prevalence	4yr prevalence
**Estimate in total sample**	14.8	BF (15.1)<br>SA (40.0)<br>Kenya (25.6)<br>Ghana (24.5)	58.4	12.1	37.0	10.3	12.5	22

Name of cohort	ACCME's HPV and Cervical Cancer Study	AWI-Gen	HAALSI	NCD Intervention Programme	DUCS-HTN	Ibadan Study of Aging	Prospective Cohort in Democratic Republic of Congo	The Benin Study
**Associated risk factor(s) reported**	none reported	age, sex	age, sex	age, ethnicity, 2h plasma glucose, BMI	obesity, physical inactivity, alcohol use	sex, urban/semi-urban, overweight/ obesity	none reported	none reported
**Diabetes definition**	**diabetes** defined as yes/no	**diabetes** defined as fasting blood glucose >7.0mmol/L and/or receiving treatment for diabetes	**diabetes** defined as self-reported history and/or fasting blood glucose >7.0mmol/L and/or random blood glucose ≥11.1 and/or receiving treatment for diabetes	**diabetes** defined as self-reported history and/or fasting blood glucose >7.0mmol/L and/or 2h glucose ≥11.1 and/or receiving treatment for diabetes	**history of diabetes** defined as yes/no; using antidiabetic agents; using insulin	**diabetes** defined as self-reported history or receiving treatment for diabetes	**diabetes** defined as self-reported history and/or fasting blood glucose >7.0mmol/L and/or 2hglucose ≥11.1 and/or receiving treatment for diabetes	**high fasting glycaemia** defined as fasting blood glucose ≥5.6mmol/L
**Source of measurement**	**glucose** measured by testing spot urine samples with Multistix 10 SG reagent strip	**fasting blood glucose** measurement not reported	**blood glucose** collected from finger prick (CareSens N) and measured using CareSense N Monitor	**fasting blood glucose** measured using YSI glucose analysers within 3h of collection **OGTT** status determined according to 1999 WHO criteria	questionnaire	questions from US Health Interview Survey	**fasting blood glucose** analysed with glucometer (Ascnecia Elite) that adjusts capillary measurements to plasma values **OGTT** classification using WHO criteria	**fasting blood glucose** analysed using Elitechgroup kits
**# of time points measured**	1	1	≥8	3	1	1	8	3
**Reporting of chronic NCD**	baseline prevalence	not reported	prevalence	incidence	prevalence	12mo prevalence	incidence	4yr prevalence
**Estimate in total sample**	2		11	15.9	6	2	29/1,000pys	20

Name of cohort	ACCME's HPV and Cervical Cancer Study	AWI-Gen	HAALSI	NCD Intervention Programme	DUCS-HTN	Ibadan Study of Aging	Prospective Cohort in Democratic Republic of Congo	The Benin Study
**Associated risk factor(s) reported**	none reported		age, sex	year of study, sex, ethnicity, obesity, BP, lipids	none reported	urban, high SES	age, physical inactivity, non-diabetic hyperglycaemia, metabolic syndrome	none reported
**Obesity definition**	**obesity** definition not reported	**obesity** defined as BMI ≥30	**obesity** defined according to WHO: obese Grade I 30.0-34.9; obese Grade II 35.0-39.9; extreme obesity ≥40	**obesity** defined as BMI ≥30	**obesity** defined as obese Grade I 30-35; obese Grade II >35	**obesity** not measured	**obesity** defined using IDF Europe criteria as WC >94cm in men and >80cm in women	**obesity** defined as BMI ≥30 **abdominal obesity** defined as ≥80cm for females and ≥94cm for males
**Source of measurement**	**weight** and **height** collected	not reported	**weight** measured to nearest 0.1kg using Genesis Growth Management Scale **height** measured to nearest 0.1cm	not reported	**weight** measured using digital scale (Seca) to nearest 0.1Kg with participants wearing light clothing **height** measured to the nearest 1cm with participants not wearing shoes		**WC** measured with flexible tape to nearest 1mm with participants standing, heels together	**weight** measured in sitting position **height** measured in standing position **WC** measured in standing position
**# of time points measured**	1	1	1	1	2		1	3
**Reporting of chronic NCD**	not reported	not reported	prevalence	not reported	prevalence		prevalence	4yr prevalence
**Estimate in total sample**			Grade I (17.7) Grade II (7.4) extreme (4.9)		Grade I (19) Grade II (10)		17	obese (24.3) abdominal (42.3)

Name of cohort	ACCME's HPV and Cervical Cancer Study	AWI-Gen	HAALSI	NCD Intervention Programme	DUCS-HTN	Ibadan Study of Aging	Prospective Cohort in Democratic Republic of Congo	The Benin Study
Associated risk factor(s) reported			none reported		none reported		none reported	none reported
CKD definition	**CKD** defined as yes/no	**CKD** not measured	**CKD** not measured	**CKD** not measured	**CKD** not measured	**CKD** not measured	**CKD** not measured	**CKD** not measured
Source of measurement	spot urine samples collected and tested with Multistix 10 SG reagent strip							
# of time points measured	1							
Reporting of chronic NCD	baseline prevalence							
Estimate in total sample	0							
Associated risk factor(s) reported	none reported							
CKD definition	**CC** defined using histology	**CC** not measured	**CC** not measured	**CC** not measured	**CC** not measured	**CC** not measured	**CC** not measured	**CC** not measured
Source of measurement	pelvic examinations; colposcopy and biopsy for all with persistent hrHPV infection and CIN2+ lesion							
# of time points measured	5							
Reporting of chronic NCD	not reported							
Estimate in total sample								

Name of cohort	ACCME's HPV and Cervical Cancer Study	AWI-Gen	HAALSI	NCD Intervention Programme	DUCS-HTN	Ibadan Study of Aging	Prospective Cohort in Democratic Republic of Congo	The Benin Study
**Associated risk factor(s) reported**								

BP: blood pressure; WC: waist-circumference; OGTT: oral glucose tolerance test; CC: cervical cancer; IDF: International Diabetes Federation; ART: antiretroviral therapy

STable 6d: Characteristics of chronic NCD data of included studies with adults aged >18 years, sampled from occupational and clinical populations

Name of cohort	Basotho Gold Miners Cohort	TLC Cohort Study	Cohort of HIV-infected, ART-naïve adults	Operational cohort of treatment naïve HIV-infected men and women	Prospective cohort of nulliparous women	ABC-DO Study	HARP Study	CoLTART Study
**Hypertension definition**	**hypertension** not measured	**hypertension** not measured	**hypertension** not measured	**hypertension** not measured	**hypertension** defined as SBP ≥140 and/or DBP ≥90 **gestational hypertension** defined as hypertension that develops after 20th gestation wk without proteinuria and if returns to normal in postpartum **pre-eclampsia** defined as both hypertension and proteinuria	**high BP** defined as yes/no	**hypertension** not measured	**hypertension** defined as SBP ≥140 and/or DBP ≥90 and/or self-reported use of antihypertensive medication and/or previous diagnosis by physician
**Source of measurement**					**BP** measurement not reported **proteinuria** measured from 24hr urine specimen or semi-quantitative dip stick	questionnaire		**BP** measured using standard Omron M6
**# of time points measured**					2	1		3

Name of cohort	Basotho Gold Miners Cohort	TLC Cohort Study	Cohort of HIV-infected, ART-naïve adults	Operational cohort of treatment naïve HIV-infected men and women	Prospective cohort of nulliparous women	ABC-DO Study	HARP Study	CoLTART Study
Reporting of chronic NCD					incidence of hypertension	prevalence at baseline		baseline prevalence
Estimate in total sample					29	not reported		23.1
Associated risk factor(s) reported					cohabitation, previous abortions	ethnicity, age, BMI, alcohol intake, smoking, diabetes		protease inhibitor containing regimens
Diabetes definition	**diabetes** not measured	**diabetes** not measured	**diabetes** not measured	**diabetes** not measured	**diabetes** not measured	**diabetes** defined as yes/no	**diabetes** not measured	**diabetes** defined as self-reported history and/or fasting blood glucose ≥6.4mmol/L and/or 2h glucose ≥11.1 and/or receiving treatment for diabetes
Source of measurement						questionnaire		**fasting blood glucose** measured using Cobas Integra 400 plus
# of time points measured						1		3
Reporting of chronic NCD						prevalence at baseline		baseline prevalence
Estimate in total sample						not reported		4
Associated risk factor(s) reported						ethnicity, age, BMI, high BP		age

Name of cohort	Basotho Gold Miners Cohort	TLC Cohort Study	Cohort of HIV-infected, ART-naïve adults	Operational cohort of treatment naïve HIV-infected men and women	Prospective cohort of nulliparous women	ABC-DO Study	HARP Study	CoLTART Study
Obesity definition	**obesity** not measured	**obesity** not measured	**obesity** not measured	**obesity** defined as BMI≥30	**obesity** not measured	**obesity** defined as BMI≥30	**obesity** not measured	**abdominal obesity** defined asWHR ≥0.95 for males and ≥0.85 for females or WC ≥94cm for males and ≥80cm for females
Source of measurement				**weight** measurement not reported **height** measurement not reported		**weight** measurement not reported **height** measurement not reported		**weight** measured using Seca digital scale **height** measured using portable Seca 213 Leicester stadiometer **WC** and **HC** measured using non-stretchable Seca 201 Ergomomic Circumference Measuring tape
# of time points measured				≥15		1		3
Reporting of chronic NCD				baseline prevalence		prevalence at baseline		baseline prevalence
Estimate in total sample				0.18		not reported		0.526
Associated risk factor(s) reported				none reported		ethnicity, age		protease inhibitor

Name of cohort	Basotho Gold Miners Cohort	TLC Cohort Study	Cohort of HIV-infected, ART-naïve adults	Operational cohort of treatment naïve HIV-infected men and women	Prospective cohort of nulliparous women	ABC-DO Study	HARP Study	CoLTART Study
								containing regimens
**CKD definition**	**CKD** not measured	**CKD** defined eGFR as mildly (60-89ml/min), moderately (30-59ml/min), or severely reduced (<30ml/min)	**renal insufficiency** defined eGFR as mildly (60-89ml/min), moderately (30-59ml/min), or severely reduced (<30ml/min) or creatinine clearance as mild (121-150mmol/L), moderate (151-200), or severe (>200)	**CKD** not measured	**CKD** not measured	**CKD** not measured	**CKD** not measured	**abnormal renal function** defined as serum urea >11.9mmol/L or serum creatinine >109µmol/L
**Source of measurement**		**eGFR** estimated using Cockcroft-Gault	**eGFR** estimated from creatinine clearance prior to ART initiation; performed secondary analysis using MDRD and serum creatinnie levels alone					**serum urea** measured using Cobas Integra 400 plus **creatinine** measured from mid-stream urine using Roche Integra 400 plus
**# of time points measured**		14-56	1					3

Name of cohort	Basotho Gold Miners Cohort	TLC Cohort Study	Cohort of HIV-infected, ART-naïve adults	Operational cohort of treatment naïve HIV-infected men and women	Prospective cohort of nulliparous women	ABC-DO Study	HARP Study	CoLTART Study
**Reporting of chronic NCD**		48mo prevalence<br><br>mortality	prevalence					overall prevalence
**Estimate in total sample**		mild (30.4)<br>moderate (5.2)<br>severe (0)<br><br>normal (3.0/100pys)<br>mild (5.5)<br>moderate (21.9)<br>severe (0)	34					0.006
**Associated risk factor(s) reported**		nephrotoxicity	none reported					none reported
**Cancer definition**	Not measured	Not measured	Not measured	**CC** defined using 2001 Bethesda system	Not measured	**BC** defined using histological or cytological confirmation	**CC** defined using histology as 'negative' (≤CIN1) or 'positive' (CIN2+)	Not measured
**Source of measurement**				conventional pap smears performed		obtained from medical records	cervical samples collected using cytobrush, assessed clinically using VIA or Lugol's iodine; all participants referred for colposcopy	

Name of cohort	Basotho Gold Miners Cohort	TLC Cohort Study	Cohort of HIV-infected, ART-naïve adults	Operational cohort of treatment naïve HIV-infected men and women	Prospective cohort of nulliparous women	ABC-DO Study	HARP Study	CoLTART Study
**# of time points measured**				≥2		1	2	
**Reporting of chronic NCD**				baseline prevalence<br><br>progression from normal to LSIL or HSIL		Not applicable; all participants had BC at baseline	cumulative incidence	
**Estimate in total sample**				38%<br><br>4.6/100pys		NA	BF (1.2)<br>SA (5.8)	
**Associated risk factor(s) reported**				CD4<200		ethnicity, education, having never heard of BC, employment, pregnancy in past 3yrs, age, SES	ART	
**COPD definition**	**COPD** defined using GOLD staging system; Grade 0: FEV1/FVC ratio ≥0.7 and respiratory symptoms (cough and sputum ≥3mo)	**COPD** not measured	**COPD** not measured	**COPD** not measured	**COPD** not measured	**COPD** defined as yes/no	**COPD** not measured	**COPD** not measured

Name of cohort	Basotho Gold Miners Cohort	TLC Cohort Study	Cohort of HIV-infected, ART-naïve adults	Operational cohort of treatment naïve HIV-infected men and women	Prospective cohort of nulliparous women	ABC-DO Study	HARP Study	CoLTART Study
**Source of measurement**	**spriometry** measured without bronchodilation using Hands Rudolph pneumotachograph **symptom questionnaire** included duration of respiratory symptoms, medical history, past/present TB, smoking, alcohol					questionnaire		
**# of time points measured**	2					1		
**Reporting of chronic NCD**	mean decline in FEV1<br><br>mortality					not reported		
**Estimate in total sample**	91ml(67-116)<br><br><br>18							
**Associated risk factor(s) reported**	age, years of employment, history of TB, active baseline TB, silicosis at							

Name of cohort	Basotho Gold Miners Cohort	TLC Cohort Study	Cohort of HIV-infected, ART-naïve adults	Operational cohort of treatment naïve HIV-infected men and women	Prospective cohort of nulliparous women	ABC-DO Study	HARP Study	CoLTART Study
	baseline and follow-up							

eGFR: estimated glomerular filtration rate; BP: blood pressure; WC: waist-circumference; PA: physical activity; BC: breast cancer; CC: cervical cancer; IDF: International Diabetes Federation; GOLD: Global Initiative for Chronic Obstructive Lung Disease; ART: antiretroviral therapy; AJCC: American Joint Committee on Cancer; TNM: Classification of Tumour Malignancy; HIVø: HIV negative; VIA: visual inspection with acetic acid; TB: tuberculosis

STable 6d continued: Characteristics of chronic NCD data of included studies with adults aged >18 years, sampled from occupational and clinical populations

Name of cohort	Prospective cohort study in Ghana	RWISA	Prospective cohort study in Benin	UARTO cohort
Hypertension definition	**gestational hypertension** defined as hypertension that develops after 20th gestation wk without proteinuria and if returns to normal in postpartum<br>**pre-eclampsia** defined as both hypertension and proteinuria	**hypertension** defined as yes/no	**gestational hypertension** defined as BP ≥140/90 or increase in SBP>30 or DBP >15 with ≥1 other sign of severity such as convulsion or coma	**hypertension** defined as SBP ≥140 and/or DBP ≥90 one 3 consecutive visits or self-reported use of antihypertensive medication after ART initiation
Source of measurement	**BP** measurement not reported	questionnaire	**BP** measurement not reported	**BP** measured using aneroid sphygmomanometer (Welch Allyn Tycos 767)
# of time points measured	≥2	≥2	2	≥3
Reporting of chronic NCD	incidence	baseline prevalence	prevalence at 6mo<br><br>prevalence at 12mo	increase in SBP per year
Estimate in total sample	7.5	HIV+ (4.8)<br>HIVø (8.3)	8<br><br>3	9.6mmHg/year
Associated risk factor(s) reported	none reported	none reported	none reported	age, sex, overweight, CD4<100
Diabetes definition	**diabetes** not measured	**diabetes** not measured	**diabetes** not measured	**diabetes** not measured
Source of measurement				
# of time points measured				
Reporting of chronic NCD				
Estimate in total sample				
Associated risk factor(s) reported				
Obesity definition	**obesity** not measured	**obesity** not measured	**obesity** not measured	**obesity** defined as BMI ≥30

Name of cohort	Prospective cohort study in Ghana	RWISA	Prospective cohort study in Benin	UARTO cohort
Source of measurement				**weight** measured to nearest 1Kg with Seca 762 and participants wearing light clothing, no shoes **height** measured to nearest 0.1cm with Seca 206, no shoes
# of time points measured				≥3
Reporting of chronic NCD				baseline prevalence
Estimate in total sample				0.026
Associated risk factor(s) reported				none reported
CKD definition	**CKD** not measured	**CKD** not measured	**CKD** not measured	**CKD** not measured
Source of measurement				
# of time points measured				
Reporting of chronic NCD				
Estimate in total sample				
Associated risk factor(s) reported				
Cancer definition	Not measured	Not measured	Not measured	Not measured
Source of measurement				
# of time points measured				
Reporting of chronic NCD				
Estimate in total sample				
Associated risk factor(s) reported				
COPD definition	**COPD** not measured	**COPD** not measured	**COPD** not measured	**COPD** not measured
Source of measurement				
# of time points measured				
Reporting of chronic NCD				
Estimate in total sample				
Associated risk factor(s) reported				

STable 7a: Characteristics of risk factor data of included studies with children and young adults aged <20 years, sampled from general populations

Name of cohort	BT20	Seychelles Child Development Study	Lungwena Child Survival Study
**Demographic & Environmental Risk Factor(s)**			
**Education definition**	**education** definition not reported	**highest of parents' occupations** defined as non-manual skilled; intermediate skilled; other	**SES** not defined
**Source of measurement**	questionnaire	not reported	asset score initially derived based on HH assets
**# of time points measured**	5	1	1
**Occupation definition**	**occupation** definition not reported	Not measured	Not measured
**Source of measurement**	questionnaire		
**# of time points measured**	5		
**SES definition**	**SES** defined as low, middle, and high tertiles; measured using a physical asset-based household SES measurement tool	Not measured	Not measured
**Source of measurement**	questionnaire		
**# of time points measured**	2		
**Environmental Risk Factor(s)**			
**Definition**	Not measured	Not measured	Not measured
**Source of measurement**			
**# of time points measured**			
**Lifestyle Risk Factor(s)**			
**Smoking status definition**	**smoking status** definition not reported	Not measured	Not measured
**Source of measurement**	questionnaire		
**# of time points measured**	≥3		
**Alcohol use definition**	**alcohol use** definition not reported	Not measured	Not measured
**Source of measurement**	questionnaire		
**# of time points measured**	≥3		
**Poor diet definition**	**poor diet** definition not reported	**infant weaning** defined as appropriate when weaning porridges introduced only after 2mo and family foods before 7mo	Not measured
**Source of measurement**	measured from longitudinal food frequency analyses using questionnaire data on what/how often foods eaten	structured questionnaire used to interview guardian about food items given to infant	

Name of cohort	BT20	Seychelles Child Development Study	Lungwena Child Survival Study
# of time points measured	6	1	
Physical inactivity definition			
Source of measurement	Not measured	Not measured	Not measured
# of time points measured			
Breastfeeding definition	**breastfeeding** definition not reported	Not measured	**breastfeeding** infants considered "exclusively breastfed" until date they received something other than breast milk
Source of measurement	not reported		structured questionnaire used to interview guardian about food items given to infant
# of time points measured	6		1
Puberty definition	**age at menarche** for females and age of transition from Tanner 1 to Tanner 2 for males	Not measured	Not measured
Source of measurement	questionnaire		
# of time points measured	1		
Sexual behaviour definition	Not measured	Not measured	Not measured
Source of measurement			
# of time points measured			
Physiological Risk Factor(s)			
Birthweight status definition	**birthweight** definition not reported	**birthweight** child weight at birth	Not measured
Source of measurement	obtained from hospital records	obtained from medical records	
# of time points measured	1	1	
Body composition definition	**body composition** defined using whole-body fat and fat-free mass	Not measured	Not measured
Source of measurement	**DXA** measured according to OSCD using Hologic QDR 4500A		
# of time points measured	7		
Cholesterol definition	**abnormal lipids** definition not reported	Not measured	Not measured
Source of measurement	not reported		
# of time points measured	3		
Proteinuria definition	Not measured	Not measured	Not measured
Source of measurement			
# of time points measured			
HIV infection definition	**HIV infection** definition not reported	Not measured	Not measured
Source of measurement	not reported		
# of time points measured	1		

Name of cohort	BT20	Seychelles Child Development Study	Lungwena Child Survival Study
**TB infection definition**	Not measured	Not measured	Not measured
**Source of measurement**			
**# of time points measured**			
**Anaemia definition**	Not measured	Not measured	Not measured
**Source of measurement**			
**# of time points measured**			

SES: socio-economic status; HH: household; ISCD: International Society of Clinical Densitometry

STable 7b: Characteristics of risk factor data of included studies with mixed (children and adults) populations, sampled from general and clinical populations

Name of cohort	RCCS	Kiang West Longitudinal Population Study	GPC	Bukavu Observ Study	Ellisras Longitudinal Study	Mildmay Uganda Cohort	Prospective cohort study in Sudan
**Demographic & Environmental Risk Factor(s)**							
**Education definition**	**education** defined as none; primary; secondary; tertiary	**education** for those aged≥18yrs, defined as attended ≥primary school	**education** defined as less than primary; incomplete primary; primary; junior/secondary; above secondary	**education level (yrs)** defined as 0, 1-6 (some or completed primary), 7-12 (some or completed secondary), ≥13 (some or completed tertiary)	**education** defined as primary or secondary	**education** defined as high school or higher; primary or less	Not measured
Source of measurement	questionnaire	not reported	questionnaire	questionnaire	not reported	not reported	
# of time points measured	11	≥1	13	2	≥1	≥1	
**Occupation definition**	**occupation** defined as agricultural/house work; bar/restaurant; boda boda/trucking; student; trader/shop keeper; other	Not measured	Not measured	Not measured	**occupation** defined as 'yes' if employed or 'no' if not	Not measured	Not measured
Source of measurement	questionnaire				questionnaire		
# of time points measured	11				1		

Name of cohort	RCCS	Kiang West Longitudinal Population Study	GPC	Bukavu Observ Study	Ellisras Longitudinal Study	Mildmay Uganda Cohort	Prospective cohort study in Sudan
**SES definition**	Not measured	Not measured	**SES** defined as low, middle, and high tertiles of an asset index	**SES** definition not reported	Not measured	Not measured	Not measured
Source of measurement			questionnaire	measured from yearly census-like surveys			
# of time points measured			1	3			
**Environmental exposure definition**	**aflatoxin exposure** defined using AFB-Lys adducts in HIV negative participants	Not measured	**aflatoxin exposure** defined using AFB-Lys adducts in HIV negative participants	Not measured	Not measured	Not measured	Not measured
Source of measurement	randomly selected archived serum samples to analyse using Agilent 1200 HPLC-fluorescence system		randomly selected archived serum samples to analyse using Agilent 1200 HPLC-fluorescence system				
# of time points measured	4		8				
**Lifestyle Risk Factor(s)**							
**Smoking status definition**	Not measured	Not measured	**smoking status** defined using past, current and frequency	**smoking status** definition not reported	Not measured	Not measured	Not measured
Source of measurement			collected using WHO STEPwise questionnaire	collected using WHO STEPwise questionnaire			

Name of cohort	RCCS	Kiang West Longitudinal Population Study	GPC	Bukavu Observ Study	Ellisras Longitudinal Study	Mildmay Uganda Cohort	Prospective cohort study in Sudan
# of time points measured			1	3			
**Alcohol use definition**	Not measured	Not measured	**alcohol use** defined using past, current and frequency	**alcohol use** definition not reported	Not measured	**alcohol use** defined as yes; no	Not measured
Source of measurement			collected using WHO STEPwise questionnaire	collected using WHO STEPwise questionnaire		questionnaire	
# of time points measured			1	3		~42	
**Poor diet definition**	Not measured	Not measured	**insufficient fruit and veg consumption** defined as <5 servings of fruit or vegs a day **high staple consumption** defined as >75th percentile	**poor diet** definition not reported	**diet** definition not reported	Not measured	Not measured
Source of measurement			collected using WHO STEPwise questionnaire	collected using WHO STEPwise questionnaire	measured using 24hr recall; average of 2- 24h dietary intake		
# of time points measured			1	3	1		
**Physical inactivity definition**	Not measured	Not measured	Not measured	**moderate PA** defined as walking fast for ≥10 min or displacement by bicycle	**inactive PA** defined as <600MET-min/wk	Not measured	Not measured

Name of cohort	RCCS	Kiang West Longitudinal Population Study	GPC	Bukavu Observ Study	Ellisras Longitudinal Study	Mildmay Uganda Cohort	Prospective cohort study in Sudan
Source of measurement				collected using WHO STEPwise questionnaire	collected using IPAQ		
# of time points measured				3	1		
**Breastfeeding definition**	Not measured	Not measured	**breastfeeding** definition not reported	Not measured	Not measured	Not measured	Not measured
Source of measurement			not reported				
# of time points measured			16				
**Puberty definition**	Not measured	Not measured	**age at menarche** definition not reported	Not measured	**puberty** defined using Tanner rating scale pictures ranging from 1 (no development) to 5 (matured stage)	Not measured	Not measured
Source of measurement			not reported		breast development and genital/pubic hair development stages assessed by visual inspection		
# of time points measured			not reported		1		

Name of cohort	RCCS	Kiang West Longitudinal Population Study	GPC	Bukavu Observ Study	Ellisras Longitudinal Study	Mildmay Uganda Cohort	Prospective cohort study in Sudan
**Sexual behaviour definition**	**sexual behaviour** in past yr defined as sexually active; multiple partners; non-marital partners among married persons; used alcohol before sex; sex with partner outside community; consistent condom use with non-marital partner	Not measured	**sexual behaviour** definition not reported	Not measured	Not measured	**sexual behaviour** defined as sexually active or not	Not measured
Source of measurement	questionnaire		self-reported			not reported	
# of time points measured	11		18			≥1	
**Physiological Risk Factor(s)**							
**Birthweight status definition**	Not measured	**birthweight** definition not reported	Not measured	Not measured	**birthweight** definition not reported	Not measured	Not measured
Source of measurement		recorded to the nearest 10g within 72hr of birth			recorded from immunization cards		
# of time points measured		1			1		
**Body composition definition**	Not measured	**body composition** definition not reported	Not measured	Not measured	Not measured	Not measured	Not measured

Name of cohort	RCCS	Kiang West Longitudinal Population Study	GPC	Bukavu Observ Study	Ellisras Longitudinal Study	Mildmay Uganda Cohort	Prospective cohort study in Sudan
Source of measurement		measured using digital bioimpedance analysis scale (Tanita)					
# of time points measured		≥12					
**Cholesterol definition**	Not measured	Not measured	**abnormal lipids** defined as raised total-C >5.2mmol/l, low HDL-C <1.0 (males) or <1.3 (females), raised triglycerides >1.7	**abnormal lipids** definition not reported	Not measured	**lipids** defined using Total-C, HDL	Not measured
Source of measurement			**lipid** measurement not reported	**lipids** measured from 4 mL of blood		**lipid** measurement not reported	
# of time points measured			1	1		≥1	
**Proteinuria definition**	Not measured			**proteinuria** definition not reported		testing not reported	
Source of measurement				qualitative proteinuria by dipstick		≥1	
# of time points measured				1			
**HIV infection definition**	**HIV infection** defined as positive or negative		**HIV infection** defined as positive if 3 of 3 tests are positive				

Name of cohort	RCCS	Kiang West Longitudinal Population Study	GPC	Bukavu Observ Study	Ellisras Longitudinal Study	Mildmay Uganda Cohort	Prospective cohort study in Sudan
Source of measurement	finger stick for rapid testing; venous blood tested with ELISA and confirmed by western blot		1) tested using rapid testing algorithm 2) retested with 1/2 Stat-Pak 3) evaluated with Uni-Gold Recombinant HIV-1/2;				
# of time points measured	11		22				
**TB infection definition**	Not measured					**current TB infection** defined as no signs on treatmentl under investigation	
Source of measurement						testing not reported	
# of time points measured						≥1	
**Anaemia definition**	Not measured	**anaemia** defined using WHO criteria: 6-59 months <=11; 5-11yrs <=11.5; 12-14 <=12; non pregnant women <=12; pregnant women <=11; men <=13"	**anaemia** defined using the WHO criteria: mild ≥110 to <130 in males or ≥110 to <120 in females; moderate ≥80 to <110 in both; severe <80 in both				**anaemia** definition not reported
Source of measurement		**Hb** measured by Medonic M-series 3-part haemotology	**Hb** measured using Coulter AC.T 5 Diff CP analyser				**Hb levels** obtained from medical records

Name of cohort	RCCS	Kiang West Longitudinal Population Study	GPC	Bukavu Observ Study	Ellisras Longitudinal Study	Mildmay Uganda Cohort	Prospective cohort study in Sudan
		analyser or HemoCue					
# of time points measured		≥12	8				2

SES: socio-economic status; HH: household; Hb: haemoglobin; PA: physical activity; IPAQ: International Physical Activity Questionnaire; TB: tuberculosis

STable 7c: Characteristics of risk factor data of included studies with adults aged >18 years, sampled from general populations

Name of cohort	ACCME's HPV and Cervical Cancer Study	AWI-Gen	HAALSI	NCD Intervention Programme	DUCS-HTN	Ibadan Study of Aging	Prospective Cohort in Democratic Republic of Congo	The Benin Study
**Demographic & Environmental Risk Factor(s)**								
**Education definition**	**education** defined as no formal schooling; primary; secondary; university; postgraduate	**education** definition not reported	**education** defined as no formal schooling; primary; some secondary; secondary or more	**education** defined as ≤3yrs; 4-9yrs; ≥10yrs	**education** defined as none; ≥some primary; ≥some secondary	Not measured	Not measured	**education** defined as no schooling; primary school; high school
Source of measurement	questionnaire	questionnaire	questionnaire	questionnaire	questionnaire			questionnaire
# of time points measured	1	1	≥8	≥1	1			≥1
**Occupation definition**	**occupation** defined as professional; self-employed; manual; skilled manual; student; unemployed	**occupation** definition not reported	**occupation** defined as employed; unemployed; homemaker	Not measured	**occupation** defined as retired/unemployed; self-employed; employed	Not measured	Not measured	Not reported
Source of measurement	questionnaire	questionnaire	questionnaire		questionnaire			
# of time points measured	1	1	≥8		1			

Name of cohort	ACCME's HPV and Cervical Cancer Study	AWI-Gen	HAALSI	NCD Intervention Programme	DUCS-HTN	Ibadan Study of Aging	Prospective Cohort in Democratic Republic of Congo	The Benin Study
**SES definition**	**SES** defined using wealth index as lowest 40%, middle 40%, and highest 20%	**SES** defined using HH attributes	**SES** defined using wealth index quintiles	**SES** defined using HH monthly income into low (<2,000 Mauritian Rupee), middle (2,500-4,999), high (≥5,000)	**SES** defined as HH wealth index quintiles: richest; richer; average; poorer; poorest	**SES** defined as low if ratio of participant's total possessions to median number of poesssions in total sample is <0.5, low-average if 0.5-1.0, high-average if 1.0-2.0, high if >2.0	**SES** defined as high/low	**SES** defined as low; medium; high
Source of measurement	generated wealth index using PCA with varimax rotation to compute factor scores based on sum of ownership of HH items weighted by factor loading	questionnaire	questionnaire	questionnaire	created through PCA of HH characteristics and assets	assessed by taking inventory of 21 HH and personal items	questionnaire	assessed using HH amenity score as proxy for HH income
# of time points measured	1	1	≥8	≥1	1	1	1	≥1
**Lifestyle Risk Factor(s)**								
**Smoking status definition**	**smoking status** defined as age when use became regular; measure of use/daily and tutal duration;	**smoking status** definition not reported	**smoking status** defined as past/present; quantity; frequency; duration; type	**smoking status** defined as yes/no	**smoking status** defined as never; former; current, 0-9/day; current, ≥10/day	**smoking status** defined as ever smoked; yes; no	**smoking status** defined as current smoker/non-smoker	**smoking status** defined as current; former (stopped ≤6mo prior); non-smoker

Name of cohort	ACCME's HPV and Cervical Cancer Study	AWI-Gen	HAALSI	NCD Intervention Programme	DUCS-HTN	Ibadan Study of Aging	Prospective Cohort in Democratic Republic of Congo	The Benin Study
	preferred type/brand; start or successfully quit; exposure to second hand smoke							
Source of measurement	tools adapted from PhenX toolkit version 5.6	questionnaire	questionnaire	questionnaire	questionnaire	questionnaire	questionnaire	collected using WHO-STEPwise questionnaire
# of time points measured	≥1	1	≥8	≥1	1	2	1	≥1
**Alcohol use definition**	**alcohol use** defined as age when use became regular; measure of use/daily and tutal duration; preferred type/brand; start successfully quit	**alcohol use** definition not reported	**alcohol use** defined as ever consumed; daily quantity; type; binge drinking behaviours	**alcohol use** defined as yes/no	**alcohol use** defined as 0g/day; 0-10; >10	**alcohol use** defined as ever drank; yes; no	**alcohol use** defined as non-drinker/drinker	**alcohol use** defined as none (0g/day); moderate (≤15 for females, ≤20 for males); heavy (>15 for females, >20 for males)
Source of measurement	tools adapted from PhenX toolkit version 5.6	questionnaire	questionnaire	questionnaire	questionnaire; assumed 14g as standard drink portion size	questionnaire	questionnaire	collected using WHO-STEPwise questionnaire
# of time points measured	≥1	1	≥8	≥1	1	2	1	≥1

Name of cohort	ACCME's HPV and Cervical Cancer Study	AWI-Gen	HAALSI	NCD Intervention Programme	DUCS-HTN	Ibadan Study of Aging	Prospective Cohort in Democratic Republic of Congo	The Benin Study
**Poor diet definition**	**diet** definition not reported	**diet** definition not reported	**diet** defined as consumption, frequency, quantity of fruit, vegetables, bread, soft drinks	Not measured	Not measured	Not measured	**diet** defined as Western-style breakfast with bread, tea and milk, butter, margarine, eggs, animal fat and protein vs African traditional diet with cassaca or maize and vegetables	**diet** defined as low, medium, high food score
Source of measurement	Nigerian food frequency questionnaire previously developed by study investigators	questionnaire	questionnaire				questionnaire	sentinel food consumption score calculated from short food frequency questionnaire of 10 food groups in last 3mo
# of time points measured	1	1	≥8				1	2
**Physical inactivity definition**	**PA** definition not reported	**PA** definition not reported	**PA** defined as type of work, excerise and sedentary activity; amount of time spent	Not measured	**PA** defined as highest (144.0-1008.0MET/hrs/wk); higher (47.0-141.3); average (14.7046.7); lower (4.4-14.0); lowest (0.0-4.0)	Not measured	**physical inactivity** defined by both 75th percentiles of seated and laying position time in hrs and MET value ranging 3-6kcal/min	**physical inactivity** defined as ≥3 MET <30min or <3 MET, any duration

Name of cohort	ACCME's HPV and Cervical Cancer Study	AWI-Gen	HAALSI	NCD Intervention Programme	DUCS-HTN	Ibadan Study of Aging	Prospective Cohort in Democratic Republic of Congo	The Benin Study
Source of measurement	modified Harvard School of Public Health's Nurses' Health Study II PA questionnaire	exercise/general PA questionnaire	International PA Questionnaire		collected using GPAQ to assess PA for work, transportation, leisure		collected using GPAQ with Comparative Risk Asessment methodology	collected using WHO-STEPwise questionnaire
# of time points measured	≥2	1	≥8		1		1	≥1
**Breastfeeding definition**	Not measured	Not measured	Not measured	Not measured	Not measured	Not measured	Not measured	Not measured
Source of measurement								
# of time points measured								
**Puberty definition**	**puberty** defined as age start of regular periods	Not measured	Not measured	Not measured	Not measured	Not measured	Not measured	Not measured
Source of measurement	questionnaire							
# of time points measured	5							
**Sexual behaviour definition**	**sexual behaviour** defined as history of sexual intercourse (vaginal, anal, oral), use of contraceptive, sex toys/lubcricants	Not measured	**sexual behaviour** definition not reported	Not measured	Not measured	Not measured	Not measured	Not measured
Source of measurement	questionnaire		questionnaire					

Name of cohort	ACCME's HPV and Cervical Cancer Study	AWI-Gen	HAALSI	NCD Intervention Programme	DUCS-HTN	Ibadan Study of Aging	Prospective Cohort in Democratic Republic of Congo	The Benin Study
# of time points measured	2		≥1					
**Physiological Risk Factor(s)**								
**Birthweight status definition**	Not measured	Not measured	Not measured	Not measured	Not measured	Not measured	Not measured	Not measured
Source of measurement								
# of time points measured								
**Body composition definition**	Not measured	Not measured	Not measured	Not measured	Not measured	Not measured	Not measured	Not measured
Source of measurement								
# of time points measured								
**Cholesterol definition**	**abnormal lipids** defined as yes/no	**abnormal lipids** defined using HDL, LDL, Total-C, Tg	**abnormal lipids** defined as questionnaire history; measured Total-C ≥6.2mmol/L or LDL>4.1 or HDL <1.19 or Tg >2.25 or taking medication for condition	**abnormal lipids** defined using HDL, Total-C, Tg	Not measured	Not measured	**abnormal lipids** defined using Tg and HDL	**abnormal lipids** defined as Tg >1.70mmol/L, HDL ≤1,29 in females and ≤1.03 in males
Source of measurement	**lipid** measurement not reported	**lipids** measured from fasting blood samples	**lipids** measured using finger prick PTS Panel #1710 lipid panel test strips	**lipids** measured using fasting blood samples analysed with manual enzymatic	Not measured	Not measured	**lipids** measured using fasting serum and plasma samples collected, frozen	**lipids** measured using fasting blood samples collected, frozen at -30C, and

Name of cohort	ACCME's HPV and Cervical Cancer Study	AWI-Gen	HAALSI	NCD Intervention Programme	DUCS-HTN	Ibadan Study of Aging	Prospective Cohort in Democratic Republic of Congo	The Benin Study
			(Cardio Chek PA Silver version)	methods (1987) and Chemistry Profile Analyzer Model LS (Coultronics) (1992,1998)			at -20C, and analysed	analysed with Elitechgroup kits
# of time points measured	2	1	≥8	2			1	3
**Proteinuria definition**	**proteinuria** definition not reported	Not measured	Not measured	Not measured	Not measured	Not measured	Not measured	Not measured
Source of measurement	spot urine samples collected and tested with Multistix 10 SG reagent strip							
# of time points measured	1							
**HIV infection definition**	Not measured	**HIV infection** definition not reported	**HIV infection** defined as positive if confirmatory test was positive	Not measured	Not measured	Not measured	Not measured	Not measured
Source of measurement		testing offered to participants on voluntary basis	determined 1) Vironostika Unifrom 11 screening assay; 2) if positive, confirmed using Roche Elecys					
# of time points measured		1	≥8					

Name of cohort	ACCME's HPV and Cervical Cancer Study	AWI-Gen	HAALSI	NCD Intervention Programme	DUCS-HTN	Ibadan Study of Aging	Prospective Cohort in Democratic Republic of Congo	The Benin Study
**HPV infection definition**	**HPV-DNA** defined as HPV positive/HPV negative	Not measured	Not measured	Not measured	Not measured	Not measured	Not measured	Not measured
Source of measurement	Cervical exfoliated samples anlaysed for HPV and hrHPV using SPF10 PCR-DEIA-LIPA25, version 1							
# of time points measured	2							
**TB infection definition**	Not measured	Not measured	**TB infection** definition not reported	Not measured	Not measured	Not measured	Not measured	Not measured
Source of measurement			questionnaire					
# of time points measured			≥8					
**Anaemia definition**	Not measured	Not measured	**anaemia** defined as mild 12.9g/dl-11 in males and 11.9-11 in females; moderate 11-8 in both; severe <8 in females	Not measured	Not measured	Not measured	Not measured	Not measured
Source of measurement			**Hb** measured using finger prick Hemocue Hb 201					

Name of cohort	ACCME's HPV and Cervical Cancer Study	AWI-Gen	HAALSI	NCD Intervention Programme	DUCS-HTN	Ibadan Study of Aging	Prospective Cohort in Democratic Republic of Congo	The Benin Study
			+microvette (Hemocue Hb 201 +Analyser)					
# of time points measured			≥8					
**Genetics**	Not measured	**genetics** data held in genome-wide SNP genotype database	Not measured	Not measured	Not measured	Not measured	Not measured	Not measured
Source of measurement		H3Africa SNP array						
# of time points measured		1						

Hb: haemoglobin; PA: physical activity; TB: tuberculosis

STable 7d: Characteristics of risk factor data of included studies with adults aged >18 years, sampled from occupational and clinical populations

**Name of cohort**	**Basotho Gold Miners Cohort**	**TLC Cohort Study**	**Cohort of HIV-infected, ART-naïve adults**	**Operational cohort of treatment naïve HIV-infected men and women**	**Prospective cohort of nulliparous women**	**ABC-DO Study**	**HARP Study**	**CoLTART Study**
**Demographic & Environmental Risk Factor(s)**								
**Education definition**	Not measured	**education** defined as no formal education; secondary; tertiary	Not measured	Not measured	**education** defined as none; primary; secondary; tertiary	**education** defined as <secondary/>secondary	**education** defined as no schooling; primary/incomplete secondary; completed secondary; graduate/postgraduate; not known	**education** defined as incomplete primary/none; complete primary; ≥secondary
Source of measurement		questionnaire			questionnaire	questionnaire	questionnaire	questionnaire
# of time points measured		1			1	1	1	1
**Occupation definition**	Not measured	**occupation** defined as unemployed/employed	Not measured	**occupation** defined as employed/unemployed	Not measured	**occupation** defined as skilled; unskilled; n/a	**occupation** defined as employed/unemployed	**occupation** defined as peasant farmer; gainful employment; self-employed/business; unemployed
Source of measurement		questionnaire		questionnaire		questionnaire	questionnaire	questionnaire
# of time points measured		1		≥15		1	1	1

Name of cohort	Basotho Gold Miners Cohort	TLC Cohort Study	Cohort of HIV-infected, ART-naïve adults	Operational cohort of treatment naïve HIV-infected men and women	Prospective cohort of nulliparous women	ABC-DO Study	HARP Study	CoLTART Study
**SES definition**	Not measured			**SES** defined using HH income as <100 Rand/mo; 1000-5000; >5000		**SES** defined using sum of 9 equally weighted possessions as low (0-3), medium (4-6), high (7-9)		**SES** defined using HH asset score tertiles
Source of measurement				questionnaire		questionnaire		questionnaire
# of time points measured				1		1		1
**Environmental exposure definition**	**silica dust exposure** defined as no dust; low dust; medum dust; high dust	Not measured	Not measured	Not measured	Not measured	Not measured	Not measured	Not measured
Source of measurement	classified by company occupational hygienest using data from mine's routine dust surveillance programme and miners' last jobs							
# of time points measured	2							
**Lifestyle Risk Factor(s)**								

Name of cohort	Basotho Gold Miners Cohort	TLC Cohort Study	Cohort of HIV-infected, ART-naïve adults	Operational cohort of treatment naïve HIV-infected men and women	Prospective cohort of nulliparous women	ABC-DO Study	HARP Study	CoLTART Study
**Smoking status definition**	**smoking status** defined as yes/no	Not measured	Not measured	Not measured	Not measured	**smoking status** defined as ever/never regularly smoked (>5 cig/day or use of snuff/wk for 6 mo)	**smoking status** defined as never/ever	**smoking status** defined as never; ex-smoker; current
Source of measurement	questionnaire					questionnaire	questionnaire	questionnaire
# of time points measured	2					1	1	1
**Alcohol use definition**	Not measured	Not measured	Not measured	Not measured	Not measured	**alcohol intake** defined as ever drank, now or in past; if yes, how often per wk	**alcohol use** defined as never; sometimes but <monthly; ≥monthly	**alcohol use** defined as never; ever >1mo prior; within <1mo
Source of measurement						questionnaire	questionnaire	questionnaire
# of time points measured						1	1	1
**Poor diet definition**	Not measured	Not measured	Not measured	Not measured	Not measured	Not measured	Not measured	**diet** defined as consumption of animal protein, fruit, vegetables, sugar, salt
Source of measurement								questionnaire
# of time points measured								1

Name of cohort	Basotho Gold Miners Cohort	TLC Cohort Study	Cohort of HIV-infected, ART-naïve adults	Operational cohort of treatment naïve HIV-infected men and women	Prospective cohort of nulliparous women	ABC-DO Study	HARP Study	CoLTART Study
**Physical inactivity definition**	Not measured	Not measured	Not measured	Not measured	Not measured	Not measured	Not measured	**PA** defined as low; moderate; high
Source of measurement								questionnaire
# of time points measured								1
**Breastfeeding definition**	Not measured	Not measured	Not measured	Not measured	Not measured	**breastfeeding** defined as yes/no; if yes, for how long	Not measured	Not measured
Source of measurement						questionnaire		
# of time points measured						1		
**Puberty definition**	Not measured	Not measured	Not measured	Not measured	Not measured	**puberty** defined as age at first menstruation	Not measured	Not measured
Source of measurement						questionnaire		
# of time points measured						1		
**Sexual behaviour definition**	Not measured	Not measured	Not measured	Not measured	**sexual behaviour** defined using condom use as never; ever	Not measured	**sexual behaviour** defined as age at fist sex; lifetime sexual partners; ever cleans vagina; # of partners in last	Not measured

Name of cohort	Basotho Gold Miners Cohort	TLC Cohort Study	Cohort of HIV-infected, ART-naïve adults	Operational cohort of treatment naïve HIV-infected men and women	Prospective cohort of nulliparous women	ABC-DO Study	HARP Study	CoLTART Study
							3mo; condom use	
Source of measurement					questionnaire		1	
# of time points measured					1		≥1	
**Physiological Risk Factor(s)**								
**Birthweight status definition**	Not measured	Not measured	Not measured	Not measured	Not measured	Not measured	Not measured	Not measured
Source of measurement								
# of time points measured								
**Body composition definition**	Not measured	Not measured	Not measured	Not measured	Not measured	Not measured	Not measured	Not measured
Source of measurement								
# of time points measured								
**Cholesterol definition**	Not measured	Not measured	Not measured	Not measured	Not measured	Not measured	Not measured	**abnormal lipids** defined as HDL<1, LDL>3.4, Total-C>5.2, TG>1.69, or Total-C-HDL

**Name of cohort**	**Basotho Gold Miners Cohort**	**TLC Cohort Study**	**Cohort of HIV-infected, ART-naïve adults**	**Operational cohort of treatment naïve HIV-infected men and women**	**Prospective cohort of nulliparous women**	**ABC-DO Study**	**HARP Study**	**CoLTART Study**
								ratio>5.1 or being on lipid lower medicine
Source of measurement								**lipids** measured using Cobas Integra 400 plus
# of time points measured								3
**Proteinuria definition**	Not measured	Not measured	**proteinuria** defined as ≥0.3g of protein or 1+ or greater	Not measured	Not measured	Not measured	Not measured	Not measured
Source of measurement			24hr urine specimen or semi-quantitative dip stick					
# of time points measured			1					
**HIV infection definition**	**HIV infection** definition not reported	Not measured	**HIV infection** defined using WHO clinical staging	Not measured	Not measured	**HIV infection** defined as yes; no/not known	**HIV infection** defined as HIV-1 serostatus	Not measured
Source of measurement	urine specimen screened using GACPAT with GACELISA to confirm		not reported			questionnaire; medical records if HIV tested	blood samples collected	

Name of cohort	Basotho Gold Miners Cohort	TLC Cohort Study	Cohort of HIV-infected, ART-naïve adults	Operational cohort of treatment naïve HIV-infected men and women	Prospective cohort of nulliparous women	ABC-DO Study	HARP Study	CoLTART Study
# of time points measured	≥1		≥2			1	1	
**HPV infection definition**	Not measured	Not measured	Not measured	Not measured	Not measured	Not measured	**HPV-DNA** defined as high risk using International Agency for Research on Cancer classification: 'carcinogenic to humans' (HPV 16, 18 , 31, 33, 35, 39, 45, 51, 52, 56, 58, 59) and 'probable carcinogenic' (HPV68**)**	Not measured
Source of measurement							cervical samples collected using Digene cervical sampler; HR-HPV testing performed using qualitative Digene hC-II; genotyping with INNO-LiPA HPV genotyping Extra assay	
# of time points measured							2	

Name of cohort	Basotho Gold Miners Cohort	TLC Cohort Study	Cohort of HIV-infected, ART-naïve adults	Operational cohort of treatment naïve HIV-infected men and women	Prospective cohort of nulliparous women	ABC-DO Study	HARP Study	CoLTART Study
**TB infection definition**	**TB infection** definition not reported	Not measured	Not measured	**TB infection** defined as current (either an diagnosis occurred within 9mo, at baseline, or within 60 days after baseline) or past (diagnosis occurred ≥9 months prior to baseline)	Not measured	**TB infection** defined as yes/no	Not measured	Not measured
Source of measurement	based on symptoms and/or chest radiography with 2 sputum samples collected from suspects for smear microscopy and liquid culture			>1 sputum sample collected from suspects for smear microscopy and liquid culture		questionnaire		
# of time points measured	2			2		1		
**Anaemia definition**	Not measured	Not measured	Not measured	Not measured	Not measured	**anaemia** defined as yes/no	Not measured	Not measured
Source of measurement						questionnaire		

Name of cohort	Basotho Gold Miners Cohort	TLC Cohort Study	Cohort of HIV-infected, ART-naïve adults	Operational cohort of treatment naïve HIV-infected men and women	Prospective cohort of nulliparous women	ABC-DO Study	HARP Study	CoLTART Study
# of time points measured						1		

PCA: principal components analysis; HH: household; PA: physical activity; Tg: triglycerides; GPAQ: Global Physical Activity Questionnaire; Hb: haemoglobin; SES: socio-economic status; Hb: haemoglobin

STable 7d continued: Characteristics of risk factor data of included studies with adults aged >18 years, sampled from occupational and clinical populations

Name of cohort	Prospective cohort study in Ghana	RWISA	Prospective cohort study in Benin	Uganda AIDS Rural Treatment Outcomes (UARTO) cohort
**Demographic & Environmental Risk Factor(s)**				
**Education definition**	**educaiton** defined as no formal education; primary; lower secondary; upper secondary; higher tertiary	**Not measured**	**education** defined as any formal education/none	**education** defined as <primary/>primary
Source of measurement	questionnaire		questionnaire	questionnaire
# of time points measured	1		3	≥1
**Occupation definition**	**occupation** defined using economic activity as informal sector employment; formally employed; non-economically active	Not measured	Not measured	**occupation** defined as employed/unemployed
Source of measurement	questionnaire			questionnaire
# of time points measured	1			≥1
**SES definition**	Not measured	**SES** defined as income <10K Rwandan Francs; 10K-35K; >35K	**SES** defined using wealth quintiles as 1 (most poor) - 5 (least poor)	**SES** defined as HH asset wealth
Source of measurement		questionnaire	questionnaire	measured by applying PCA to 25 binary variables of HH assets and characteristics
# of time points measured		1	3	≥1
**Lifestyle Risk Factor(s)**				
**Smoking status definition**	Not measured	Not measured	Not measured	**smoking status** defined as never; past/current

Name of cohort	Prospective cohort study in Ghana	RWISA	Prospective cohort study in Benin	Uganda AIDS Rural Treatment Outcomes (UARTO) cohort
Source of measurement				questionnaire
# of time points measured				≥1
**Poor diet definition**	Not measured	Not measured	Not measured	**diet** defined as average number of meals per wk with meat
Source of measurement				questionnaire
# of time points measured				1
**Breastfeeding definition**	Not measured	Not measured	Not measured	Not measured
Source of measurement				
# of time points measured				
**Puberty definition**	Not measured	Not measured	Not measured	Not measured
Source of measurement				
# of time points measured				
**Sexual behaviour definition**	Not measured	Not measured	Not measured	**sexual behaviour** defined as number of sexual partners in lifetime and incidences of rape
Source of measurement				questionnaire
# of time points measured				1
**Physiological Risk Factor(s)**				
**Birthweight status definition**	Not measured	Not measured	Not measured	Not measured

Name of cohort	Prospective cohort study in Ghana	RWISA	Prospective cohort study in Benin	Uganda AIDS Rural Treatment Outcomes (UARTO) cohort
Source of measurement				
# of time points measured				
**Body composition definition**	Not measured	Not measured	Not measured	Not measured
Source of measurement				
# of time points measured				
**Cholesterol definition**	Not measured	**abnormal lipids** definition not reported	Not measured	Not measured
Source of measurement		**lipids** measured and lipoprotein levels determined		
# of time points measured		1		
**Proteinuria definition**	Not measured	**proteinuria** defined as ≥+1	Not measured	Not measured
Source of measurement		single urine sample		
# of time points measured		1		
**HIV infection definition**	Not measured	**HIV infection** defined as postive/negative	Not measured	Not measured
Source of measurement		diagnosis performed by testing algorithm, requiring two positive results form commercial HIV-1 antibodies ELISA kits		
# of time points measured		1		

Name of cohort	Prospective cohort study in Ghana	RWISA	Prospective cohort study in Benin	Uganda AIDS Rural Treatment Outcomes (UARTO) cohort
**HPV infection definition**	**birthweight** defined as low (<2500g)	**HPV-DNA** defined as 1) positive for HPV-16; 2) negative for HPV-16, positive for HPV-18; 3) positive for other carcinogenic types but not for HPV-16, 18; 4) positive for non-carcinogenic types and negative for all carcinogenic types; 5) HPV negative by PCR	Not measured	Not measured
Source of measurement	measured within 24hr of birth	CVL specimens digested with Proteinase K, detected using the L1 MY09/MY11 modified PCR system with AmpliTaq Gold polymerase, products probed for the presence of HPV DNA by Southern blot with radiolabeled generic probe mixture		
# of time points measured	1	1		
**Anaemia definition**	Not measured	Not measured	**anaemia** defined as severe if Hb<4g/dl or if Hb <4g/dl and pallor with schock, respiratory diffulties, transfusion	Not measured
Source of measurement			**Hb** measured using HemoCue	
# of time points measured			2	
